# Exploring the boundaries between neoplastic and reactive lymphoproliferations: lymphoid neoplasms with indolent behavior and clonal lymphoproliferations—a report of the 2024 EA4HP/SH lymphoma workshop

**DOI:** 10.1007/s00428-025-04168-5

**Published:** 2025-07-05

**Authors:** Leticia Quintanilla-Martinez, Jan Bosch-Schips, Gorana Gašljević, Michiel van den Brand, Olga Balagué, Ioannis Anagnostopoulos, Maurilio Ponzoni, James R. Cook, Stefan Dirnhofer, Birgitta Sander, Camille Laurent

**Affiliations:** 1 Institute of Pathology and Neuropathology, Comprehensive Cancer Center, Eberhard-Karls-University of Tübingenand, University Hospital Tübingen, Tübingen, Germany; 2 Cluster of Excellence iFIT (EXC2180), Image-Guided and Functionally Instructed Tumor Therapies” Eberhard-Karls-University, Tübingen, Germany; 3 Department of Pathology, Hospital Universitari de Bellvitge-Bellvitge Biomedical Research Institute, L´Hospitalet de Llobregat, Barcelona, Spain; 4 Department of Pathology, Institute of Oncology Ljubljana, Ljubljana, Slovenia; 5 Medical Faculty, University of Maribor, Taborska Cesta 8, Maribor, Slovenia; 6 Department of Pathology, Radbourd University Medical Center, Nijmegen, The Netherlands; 7 Pathology-DNA Location Rijnstate Hospital, Arnhem, the Netherlands; 8 Department of Pathology, Hospital Clinic, Barcelona, Spain; 9 Institute of Pathology, Julius-Maximilians-University Würzburg, Würzburg, Germany; 10 Pathology Unit, Hematopathology Diagnostic Area, Vita-Salute San Raffaele University, San Raffaele Scientific Institute, Milan, Italy; 11 Department of Pathology and Laboratory Medicine, Cleveland Clinic, Cleveland, OH USA; 12 Institute of Medical Genetics and Pathology, University Hospital Basel, University of Basel, Basel, Switzerland; 13 Department of Laboratory Medicine, Division of Pathology, Karolinska Institute and Karolinska University Hospital, Stockholm, Sweden; 14 Department of Pathology, Cancer Institute, Toulouse University Hospital Center, University of Toulouse-Oncopole, Toulouse, France

**Keywords:** Indolent proliferations, Clonal lymphoproliferations, Intestinal indolent proliferations

## Abstract

The boundaries between neoplastic and reactive lymphoproliferations were discussed during the 2024 European Association for Haematopathology/Society for Hematopathology workshop in Dubrovnik, Croatia. Session 5 focused on indolent lymphoid neoplasms and clonal lymphoproliferations. Seventy-two cases were submitted, representing good examples of indolent lymphomas and lymphoproliferative disorders (LPD) and their diagnostic challenges. The morphologic spectrum of primary cutaneous marginal zone lymphoma/lymphoproliferation (PC-MZL/PC-MZLPD) was discussed. PC-MZL/PC-MZLPD is divided in the immunoglobulin heavy chain switched-type and non-switched-type with some clinicopathological differences. The overlapping features between PC-MZL/PC-MZLPD and PC-CD4 + T-cell LPD were highlighted. The criteria for the diagnosis of indolent T-lymphoblastic proliferation (iT-LBP) were reviewed. Indolent T-cell lymphoproliferation of the gastrointestinal tract (iT-LPD-GI) is a rare clonal, non-destructive, and non-epitheliotropic T-cell LPD occurring in adults with a male predominance. The cases submitted to the workshop revealed clinicopathological heterogeneity. Unusual features like infiltration of the complete intestinal wall, mesenteric lymph node involvement, and splenomegaly were observed. A novel group of PD1 + /CD4 + indolent cases with intestinal tropism and dissemination to blood, bone marrow, lymph node, and skin was identified. Other indolent clonal B- and T-cell LPDs were discussed including transient, clonal CD8 + T-cell proliferations, usually the result of immune-mediated cytotoxic T-cell response to virus or neoantigens, and the recently described follicle center lymphoma (FLC) of the lower female genital tract. The increasing awareness of the existence of indolent LPDs should avoid unnecessary treatments. In this report, novel findings, recommendations for diagnosis, open questions, and diagnostic challenges raised by the cases submitted to the workshop will be discussed.

**Supplementary Information:**

The online version contains supplementary material available at 10.1007/s00428-025-04168-5.

## Introduction

Indolent lymphomas and lymphoproliferative disorders (iL-LPDs) are a heterogeneous group of disorders gaining increasing recognition in the field of hematopathology [1, 2]. The development of highly sensitive diagnostic techniques, particularly mutational assays and clonality detection, has expanded this group by identifying new entities previously described as reactive processes; a good example is indolent, clonal T-cell LPD of the gastrointestinal tract (GIT) often misinterpreted as inflammatory bowel disease (IBD) [3]. Additionally, follicle center lymphoma of the lower female genital tract is an example of an indolent lymphoma often misinterpreted as diffuse large B-cell lymphoma [3, 4]. Other lymphomas might have an early “indolent” clinical phase, such as mantle cell lymphoma or follicular lymphoma in stage 1 that eventually, over time, will evolve into an overt and clinically manifest disease that requires treatment. Furthermore, characterization of certain lymphomas, such as primary cutaneous CD4 + small/medium T-cell lymphoma (PC-CD4 + T-cell LPD), has highlighted their benign behavior, and has led to reclassification as lymphoproliferative disorder [5–7]. Despite this, iL-LPDs remain a poorly defined category with no clear-cut criteria, encompassed under the vague and ambiguous term “indolent,” lacking a universally agreed-upon definition.

“Indolent” stems from Latin *indolens*, meaning “no suffering.” In medicine, it is a broad concept applied indistinctly to different perspectives on disease behavior. Specifically, in the fields of oncology and pathology, the meaning may stretch from something clinically indolent to histologically indolent. Clinically, “indolent” describes an asymptomatic disease usually requiring no treatment, for instance, duodenal-type follicular lymphoma (DFL) [8, 9]. Histologically, it refers to features of slow growth and lack of aggressiveness, such as low proliferative rate, and absence of atypia, mitoses, or necrosis. These terms are not opposites, but they represent distinct concepts. This distinction is particularly relevant in pathology, where a disease can be clinically indolent but not histologically, and vice versa. Clinical correlation is essential to determine whether a tumor qualifies as “indolent,” since aggressive-looking tumors like indolent T-lymphoblastic proliferation (iT-LBP) may present with subtle, non-specific symptoms or without symptoms [10]. Many iL-LPDs are complex and require advanced diagnostic techniques, especially to establish clonality, although clonality is not synonymous with malignancy. An accurate diagnosis of iL-LPD is critical, often supporting monitoring and a watch and wait (W&W) approach to avoid overtreatment.

The major theme of the 2024 European association for Haematopathology (EA4HP) and Society for Hematopathology (SH) workshop in Dubrovnik, Croatia was “Exploring the boundaries between neoplastic and reactive lymphoproliferations.” A session was dedicated to lymphoid neoplasms with indolent behavior and clonal lymphoproliferations. Seventy-two cases were submitted to this session, representing the many challenges in the diagnosis of clonal lymphoproliferations with indolent behavior. The cases were divided into the following thematic groups to illustrate diagnostic dilemmas and/or interesting biological features:

1. Primary cutaneous lymphomas/lymphoproliferations
2. Indolent T- or B-lymphoblastic proliferation (iT-LBP, iB-LBP)
3. Other indolent, clonal T-cell lymphoproliferative disorders
4. EBV + lymphoproliferative disorders
5. Intestinal lymphoproliferative disorders
6. Other indolent B-cell lymphoma/lymphoproliferative disorders

## Primary cutaneous lymphomas/lymphoproliferations

The classification of primary cutaneous lymphomas (PCLs) and LPDs is continuously evolving due to the integration of novel clinical, pathological and molecular information [7, 11]. Based on long-term follow-up studies with large number of patients showing that certain PCLs have a benign clinical course, despite being clonal, recent classifications have decided to down-grade several PCL to LPDs. Examples of these are PC-MZLPD, PC-CD4 + T-cell LPD, and primary cutaneous acral CD8 + T-cell LPD (PC-CD8 + T-cell LPD) [1, 2, 12]. In the workshop, 15 cases were submitted with the diagnosis of PCL/LPD, including 9 cases with the diagnosis of PC-MZL/PC-MZLPD, 4 cases with overlapping features between PC-MZLPD and PC-CD4 + T-cell LPD and 2 cases of PC-CD8 + T-cell LPD.

### Primary cutaneous marginal zone lymphoma/lymphoproliferative disorder (PC-MZLPD)

The International Consensus Classification (ICC) and the recent 5th edition of the World Health Organization (WHO) recognized PC-MZL/PC-MZLPD as a definitive entity [1, 2]. Both classifications have separated PC-MZL/PC-MZLPD from other mucosa associated lymphoid tissue (MALT) lymphomas based on its distinctive clinical behavior, histology and genetic profile [1, 2, 13]. The WHO 5th edition classification retained the name of PC-MZL, whereas the ICC favored the name PC-MZLPD (term to be used in this report) due to its excellent clinical behavior. PC-MZLPD is a very indolent disease with a 5-year survival of almost 100% that by definition has no evidence of extracutaneous disease at the time of presentation [6, 14, 15]. The disease typically occurs in adults in the fifth to sixth decade with male predominance; however, it may occur in younger patients comprising up to 10% of the cutaneous lymphomas diagnosed in children and adolescents with a median age of 16 to 17 years [16, 17]. Clinically, PC-MZLPD presents with multifocal or less commonly solitary red or violaceous papules, plaques or nodules localized preferentially on the trunk and upper-extremities [15, 18]. Cutaneous recurrences are common; however, extracutaneous dissemination (4–8%) [6, 14] or transformation to a large B-cell lymphoma occurs rarely [19]. Association with *Borrelia burgdorferi* has been reported in a minority of cases mainly in Europe and associated with IgM expression [20, 21]. Morphologically, two subtypes of PC-MZLPD have been described that differ in some histological features, immunoglobulin heavy chain (IGH) expression, and microenvironment (Table 1) [7, 15, 22]. The majority of the cases belong to the heavy chain class-switched subtype (IgG > > > IgA > > IgE), with around 40% expressing IgG4 but are not associated with systemic IgG4-related disease [23]. The class-switched subgroup involves typically the dermis, which shows prominent plasmacytic differentiation, often follicles with reactive germinal centers (GC), a CD4 + T-cell rich component with a variable number of PD1 + cells, and Th2 microenvironment. Clusters of CD123-positive plasmacytoid dendritic cells have been reported [24]. IRTA1 and CXCR3 are not expressed [22, 25]. Cutaneous recurrences are observed but extracutaneous dissemination is exceptional. In contrast, the non-class-switched subtype (approx. 10%), IgM-positive, involves more often the subcutis, has less reactive T-cell component, and has a Th1 microenvironment, the B-cells express IRTA1 and CXCR3, and extracutaneous dissemination is more likely to occur although prognosis remains excellent [7, 15]. *FAS* gene variants are found in 60% of PC-MZLPD but are uncommon in other MZL [26].

**Table 1:** Clinicopathological features of primary cutaneous marginal zone lymphoproliferative disorder

	Heavy chain class-switched type	Heavy chain non-class-switched type
Clinical course	- Indolent course - Represent around 90% of the cases - Cutaneous relapses are common - Dissemination is rare - 10% of cutaneous lymphomas in children	- Indolent course - Represent around 10% of the cases - Similar to MALT lymphomas of other sites - Dissemination occurs more often - Association with *Borrelia burgdorgeri* in European population
Skin lesions	- Multiple skin lesions in trunk and upper extremities, rarely solitary lesion - Dermal infiltration - Nodular infiltration - Plasmacytic differentiation often - Plasma cells clusters in the periphery of the infiltrate - Often follicles with reactive germinal centers - Paucity of neoplastic B-cells - T-cell-rich component with PD1 expression	- Multiple skin lesions in trunk and upper extremities, rarely solitary lesion - Predilection for subcutis infiltration - More common diffuse growth pattern - Plasmacytic differentiation rare - Diffusely scattered plasma cells - Frequent follicular colonization - B-cell predominant infiltrate - Less reactive T-cells
Immunoglobulin heavy chain expression	- IgG in 72–95% - IgG4 in 39% - IgA in 5–17% - IgE rare	- IgM
Immunophenotype	CD20 +, CD79a +, BCL2 + CD10-, BCL6-, CD23-, CD5-, cycD1- - Clusters of CD123 BPDC occur	CD20 +, CD79a +, BCL2 + CD10-, BCL6-, CD23-, CD5-, cycD1-
Microenvironment	-T-helper type 2 (Th2) - B-cells do not express IRTA1 and CXCR3	-T-helper type 1 (Th1) - B-cells express IRTA1 and CXCR3
Clonality analysis	- IGH/IGK monoclonal - Rarely monoclonal TR	- IGH/IGK monoclonal - Rarely monoclonal TR
Molecular studies	FAS mutations 60% *IGH*::*MALT1* 25% *BIRC::MALT1* and *IGH*::*FOXP1* rare Trisomy Chr. 3 and 8 in up to 20%	The same genetic alterations *MYD88* mutations rare*

*BPDC* Blastic plasmocytoid dendritic cells, *chr* chromosome, *PC-MZLPD* primary cutaneous marginal zone lymphoproliferative disorder

^*^Controversial results; some studies have not identified this mutation

**Fig. 1:**
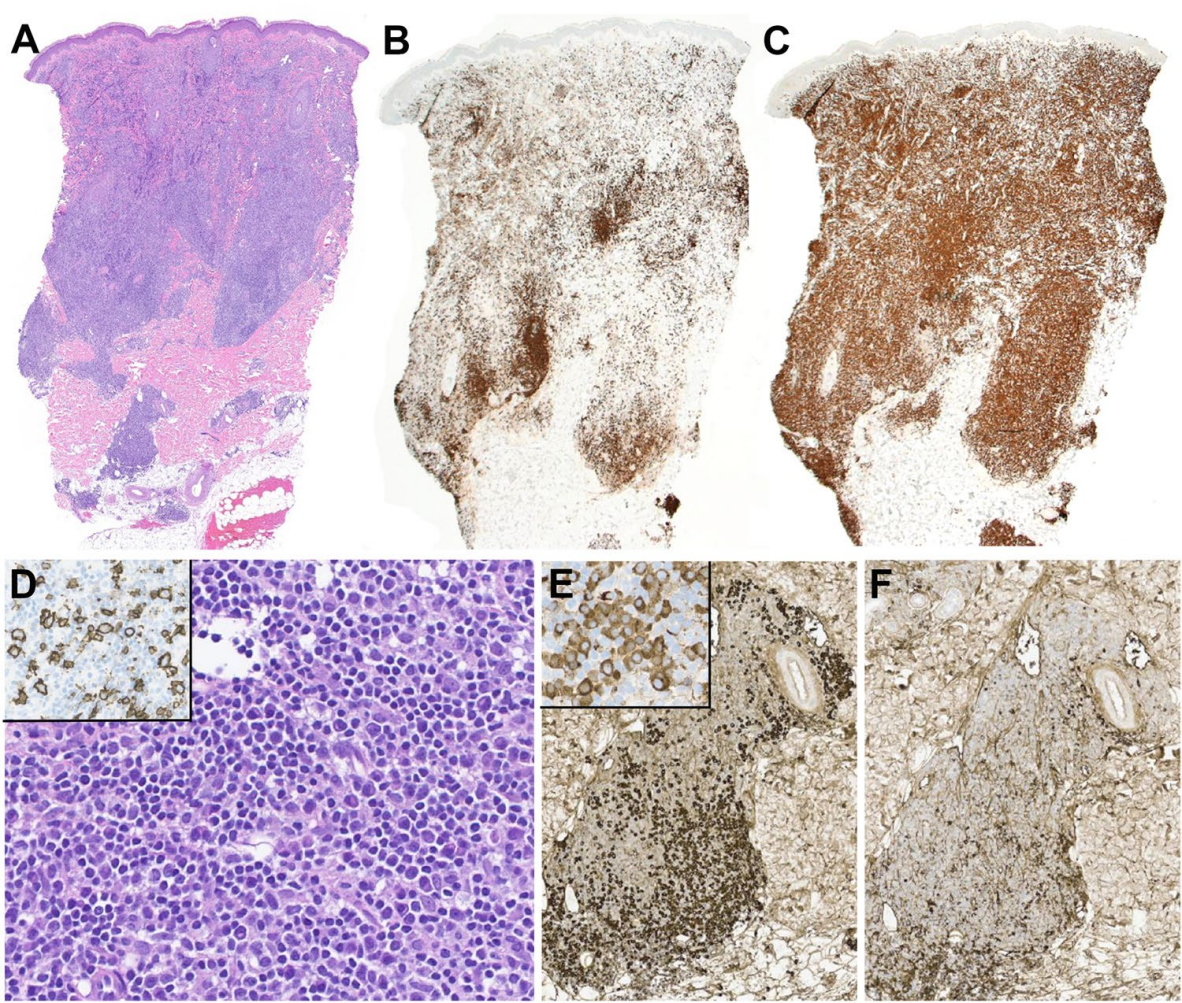
Histologic and immunophenotypic features of primary cutaneous marginal zone lymphoproliferative disorder, class-switched type in a 13-year-old boy with multiple cutaneous lesions. Case LYWS-22 courtesy of A. R. Oskardottir. **A** A panoramic view of the skin biopsy showing a dense infiltrate involving the dermis and extending into the subcutaneous tissue. Panoramic view of the skin with (**B**) CD20 and (**C**) CD3. Please note that the T-cell infiltrate predominates. The CD20 stain highlights the residual B-cell follicles present at the base of the lesion. **D** Higher magnification demonstrates that the infiltrate is polymorphic composed of small lymphocytes, plasma cells and some larger lymphoid cells. Inset: CD20 stain highlight the paucity of the B-cell infiltrate composed of large and small cells. The immunoglobulin light chain stains for (**E**) kappa and (**F**) lambda revealed monotypic kappa expression. Inset: The plasma cells express IgG

In the workshop, 6 cases were submitted with the diagnosis of PC-MZLPD (Supplemental Table 1). The cases illustrated the clinical and morphological spectrum described in the disease. There were 3 males and 3 females with a median age of 57 years (range 13–83). The case LYWS-22 presented by A.R. Oskardottir was a 13-year-old boy with multiple cutaneous lesions, who two years prior was evaluated for subcutaneous nodules involving both extremities. A biopsy from the left arm was diagnosed at the time as lymphoid hyperplasia. The present biopsy showed a dense infiltrate involving the superficial dermis, composed of small lymphocytes with abundant plasma cells (Fig. 1), and reactive follicles toward the base of the lesion. The lymphoid infiltrate was composed of CD20-positive B-cells, negative for CD10 and BCL6 with abundant reactive T-cells. The plasma cells showed restricted expression of IgG-Kappa and PCR analysis demonstrated a clonal IGH rearrangement. Despite the young age of the patient, the case represented clinically and morphologically a typical example of PC-MZLPD of class-switched type. This case also demonstrated the difficulties in the differential diagnosis with cutaneous lymphoid hyperplasia. Reactive lymphoid infiltrates of different etiologies (arthropod bites, tattoos, infectious agents, drugs or autoimmune disorders) often mimic PC-MZLPD [27]. The presence of plasma cells should prompt the analysis of light chain restriction and when possible IGH/IGK clonality analysis. In these cases, complete clinical history including any drug regimen is necessary. Moreover, the abundant reactive T-cells often seen in the class-switched type can potentially obscure the neoplastic B-cells and raise the diagnosis of a T-cell lymphoma. Two similar cases in adult patients were submitted by M. Abdulla (LYWS-296) and S. Epari (LYWS-463).

Case LYWS-115 submitted by A. Mozos was the only case of non-class-switched type submitted to the workshop. A 54-year-old woman developed a preauricular erythematous plaque without pruritus or pain. The skin biopsy showed a dense multinodular infiltrate that extended to the subcutis with occasional plasma cells and reactive GCs. The cells were CD20 + and IgM + without demonstrable light chain restriction. The patient was treated with rituximab and radiation therapy. The skin lesions recurred in several occasions during the following years. Four years later infiltration of the parotid gland and regional LN involvement was demonstrated confirming the often, extracutaneous spread of this variant. Comparative IGH clonality analysis demonstrated the same clone in the different biopsies. The non-class-switched lesions are believed to be more similar to MALT lymphomas of other sites [28].

Two additional cases submitted to the workshop represented nice examples of PC-MZLPD with special features. Case LYWS-360 submitted by J. Zhou was a 72-year-old woman that presented with 4–5 scattered papules and patches on the shoulder, chest, back, and thigh without symptoms. The skin biopsy showed a moderately dense lymphoid infiltrate within the papillary dermis with epidermotropism. The case represented a nice example of PC-MZLPD with epidermotropism [29]. This is a very rare morphological variant of PC-MZLPD. Most cases have been described in older men with disseminated skin lesions and often extracutaneous involvement including spleen and bone marrow (BM). Interestingly, despite systemic involvement, this disease does not follow an aggressive clinical course. As in this case, the B-cells characteristically express CXCR3. The case LYWS-47 presented by L. Soma represented a nice example of Epstein-Barr virus (EBV)-positive PC-MZLPD class-switched type that within 3 years transformed to a plasmablastic lymphoma with systemic dissemination. The patient was a 61-year-old man known to have psoriatic arthritis and treated with methotrexate, leflunomide, mycophenolate and sulfasalazine. The patient developed nodular skin lesions. The skin biopsy showed a nodular, predominantly dermal lymphoplasmacytic infiltrate with IgG-kappa restriction, low proliferation (< 10%) and EBER + cells. He was treated with local radiotherapy. The patient had several recurrences within the next 2 years before the transformation occurred. Molecular studies demonstrated from the first biopsy one *DNMT3A* (c.1937-1G > A; 46%) and two *TET2* (c.1133del; p.G378fs*49; 45% and c3008G < A; p.W1003*, 39%) variants interpreted as clonal hematopoiesis. At transformation, an additional *STAT3* (c.1919A.T; p.G378fs*49, 41%) variant was identified. Clonality analysis demonstrated the same IGH clone in all the biopsies. EBV-positive extranodal MZL are rare [30, 31] and develop more often in the posttransplant setting; however, they have been described in elderly patients with no specific factors (possibly due to immune senescence), and other immunodeficient conditions. EBV latency is either type I or II. In the limited genetic studies performed, no genetic alterations have been demonstrated, so far, in the cases studied. The case presented here is unusual because most patients have clinically an indolent disease with good response to reduction of immune suppression or immunochemotherapy. Aggressive transformation has not been previously reported.

Three additional cases were submitted with the diagnosis of extranodal MZL (Supplemental Table 1), two of them presented as subcutaneous tissue masses in trunk (LYWS-6 submitted by J Staniforth and LYWS-351 submitted by L.K. Aftab). The third case (LYWS-241 submitted by L. Qiu) was a 65-year-old female with a clinical history of a skin lesion diagnosed as PC-MZLPD of class-switched type treated with radiotherapy and in remission. Subsequently, she developed a conjunctival lesion with kappa-IgG4 + cells. There was insufficient material to do clonality analysis to prove the relatedness of the two neoplasms.

### Overlapping features between PC-MZLPD and PC-CD4 + T-cell LPD

**Fig. 2:**
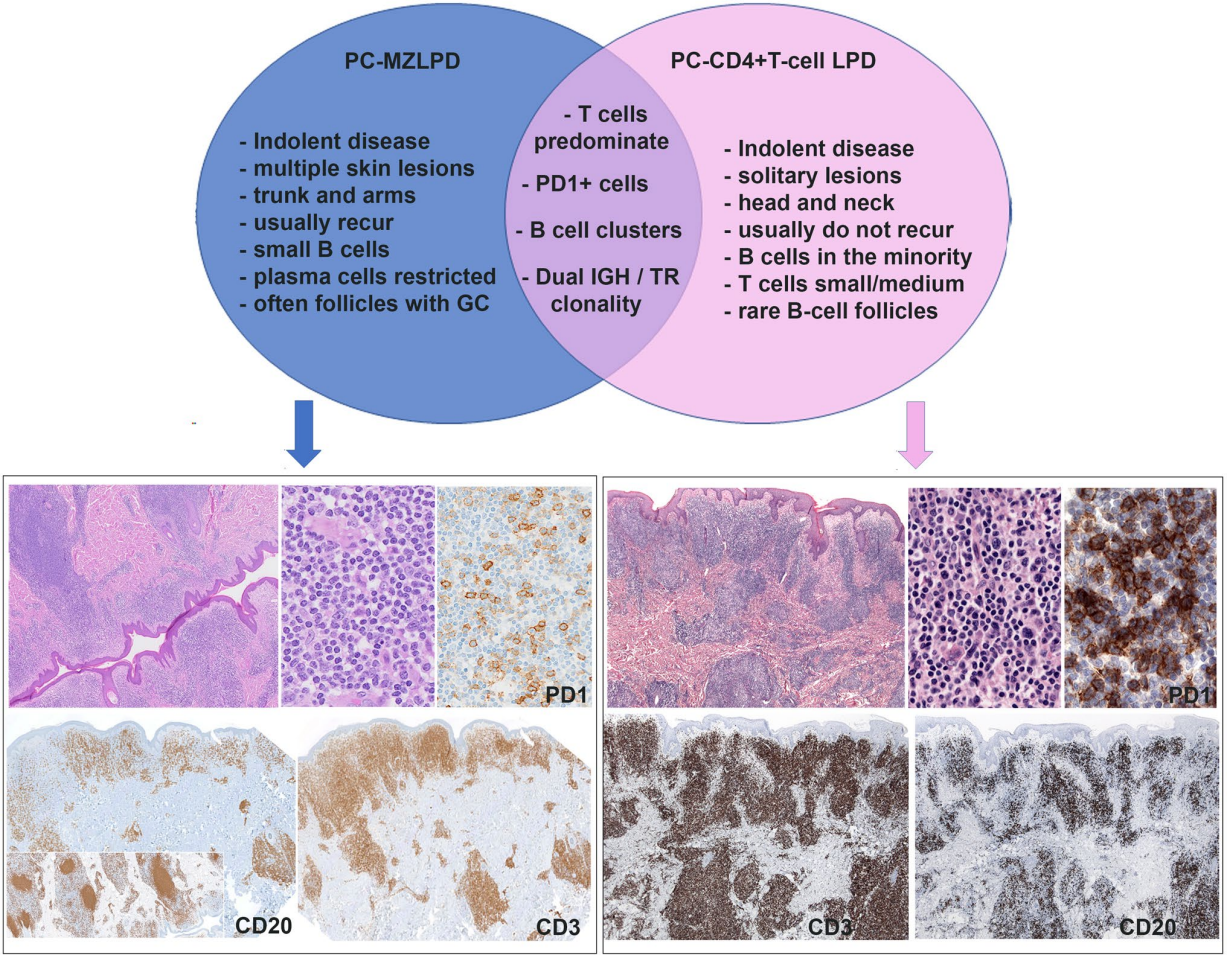
Overlapping clinical and morphologic features between primary cutaneous marginal zone lymphoproliferative disorder (PC-MZLPD) and primary cutaneous CD4 + small and medium T-cell lymphoproliferative disorder (PC-CD4 + T-cell LPD)

PC-MZLPD and PC-CD4 + T-cell LPD are two indolent lymphoproliferations with excellent prognosis and distinctive clinical, morphological and molecular features. PC-CD4 + T-cell LPD is usually a solitary lesion that typically occurs in the head and neck region or upper trunk [13]. The skin infiltrate consists of a mixture of small to medium pleomorphic CD4-positive cells with variable expression of follicular helper T-cell (TFH) markers PD1, ICOS, BCL6, and CXCL13 [32]. In most cases, there is a considerable admixture with CD20 + B-cells [33]. Despite the distinct features of PC-MZLPD and PC-CD4 + T-cell LPD, some cases exhibit morphologic, immunophenotypic and molecular overlapping features representing a real diagnostic challenge [34, 35]. Both diseases show dermal infiltrates with mixed populations of B- and T-cells, often in similar proportions. Confounding features include monotypic plasma cells in PC-CD4 + T-cell LPD, abundant PD1 + T-cells in PC-MZLPD, and dual monoclonal IGH and TR reported in both disorders that may lead to uncertainty in the diagnosis (Fig. 2) [36, 37].

**Fig. 3:**
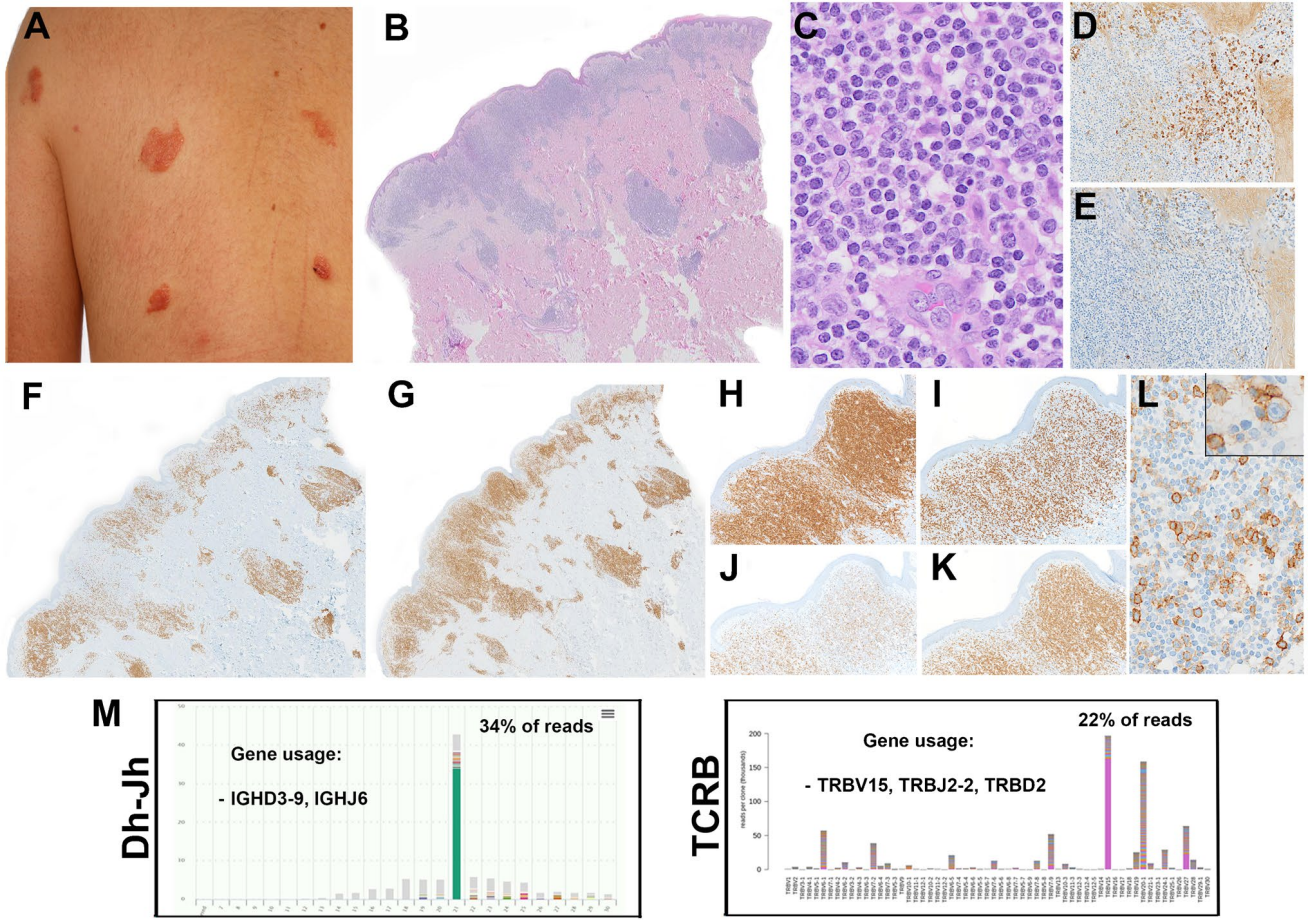
Clinical and morphologic features of a primary cutaneous lymphoproliferative disorder with overlapping features between PC-MZLPD and PC-CD4 + T-cell LPD. Case LYWS-303 courtesy of A. Vogelsberg. **A** Multiple, red papules in the shoulder and back of a 37-year-old man. **B** Panoramic view of the skin biopsy showing a dense infiltrating involving the upper dermis extending into the deep dermis in a nodular pattern. **C** Higher magnification demonstrates the monotonous, small lymphocytes infiltrate. **D, E** The immunoglobulin light chain stains demonstrate monotypic kappa expression (D) with only few, lambda positive plasma cells. **F** The CD20 stain highlights the B-cells. **G** CD3 stain reveals the predominant T-cell infiltrate. The T-cell infiltrate is predominantly **H** CD4-positive with **I** fewer CD8 positive cells. The T-cells have **J** loss/weak CD7 expression with **K** normal CD5 and CD2 expression (not shown). **L** PD1 stain demonstrates many scattered positive small and medium lymphoid T-cells. **M** NGS-based clonality analyses demonstrated dual B-cell and T-cell clones

Four cases were submitted with the diagnosis of overlapping features between PC-MZLPD and PC-CD4 + T-cell LPD (Supplemental Table 2). All were men with a median age of 43.5 years (range 37–80 years). Morphologically, all four cases showed similar features and 3/4 cases revealed dual monoclonality for IGH and TR. Case LYWS-303 presented by A. Vogelsberg was a 37-year-old man with multiple lesions in shoulder and upper back (Fig. 3). A skin biopsy showed a dense dermal lymphoid infiltrate with diffuse vaguely nodular pattern. The infiltrate was composed of small to medium-sized lymphocytes, plasma cells, histiocytes and eosinophils. Immunophenotype revealed CD20 + small aggregates and kappa-IgG4 restricted plasma cells suggestive of PC-MZLPD of class-switched type. However, CD3 demonstrated numerous T-cells, more abundant than B-cells, CD4 +, PD1 +, ICOS +, BCL6 +, and CXCL13 + (subset) with partial loss of CD7, suggestive of a PC-CD4 + T-cell LPD. Proliferation was low. Clonality analysis revealed both IGH and TR monoclonal populations. No gene variants were identified by next generation sequencing (NGS). Because of the clinical presentation and morphological features, a diagnosis of PC-MZLPD was favored. The other two cases submitted with dual clonality (LYWS-290 submitted by J. Szakonovi and LYWS-471 submitted by R. Rolim), presented clinically with a solitary lesion, one in the head and the other in the forearm favoring the diagnosis of PC-CD4 + T-cell LPD. The fourth case (LYWS-192 submitted by I. Obiorah) was diagnosed as PC-MZLD class-switched type (IgG/IgG4) rich in TFH cells. In this case only IGH was clonal. Importantly, these cases should not be diagnosed as composite lymphomas. Clonal T-cell proliferations in B-cell lymphomas can occur as part of a normal immune response, and are usually antigen driven [38, 39]. Accordingly, it has been suggested that the overlapping features might be the result of a common etiology, probably an exaggerated immune response to an unknown antigen [40].

### Primary cutaneous acral CD8 + T-cell lymphoproliferative disorder (PC-CD8 + T-cell LPD)

Two cases were submitted with this diagnosis (LYWS-167 submitted by R. Shao and LYWS-229 submitted by T. Irwin). Both cases represented good examples of the disease (Supplemental Table 2). Case LYWS-229 was a 41-year-old man with a typical solitary lesion in the helix of the ear, presenting as a slowly growing reddish nodule. The biopsy showed a non-epidermotropic dermal lymphoid infiltrate sparing adnexal structures. The lymphoid cells were CD3- and CD8-positive with low proliferation. Additionally, the CD68 stain showed the characteristic Golgi-like positivity useful to support the diagnosis. Despite TR clonality, the prognosis is excellent. Although cutaneous relapses are reported, extracutaneous dissemination is extremely rare [7].

## Indolent T- or B-lymphoblastic proliferation and mimickers

Indolent T-lymphoblastic proliferation (iT-LBP) refers to the presence of a non-clonal, non-neoplastic population of immature T-lymphoblasts proliferating in extrathymic localization [10, 41, 42]. iT-LBP might infiltrate LN and other organs either as small clusters or sheets of cells raising the differential diagnosis with T-lymphoblastic lymphoma, ectopic thymus and thymoma. The absence of thymic epithelium helps to exclude the latter two entities. The differential diagnosis from T-lymphoblastic lymphoma/leukemia (T-LBL) might be more challenging. Criteria that favor the diagnosis of iT-LBP include lack of cytologic atypia, a TdT +/CD3 + phenotype without aberrant antigen expression and a polyclonal TR (Table 2). The lack of LMO2 expression, a marker of T-LBL, might be helpful in difficult cases [43]. iT-LBP is identified in 55% of the cases in LN, where the normal architecture is usually preserved. It has been also identified in liver (20%), GIT (11%), parotid gland (4.5%), and other organs. iT-LBP within LN is frequently described in association with Castleman disease (CD) with or without follicular dendritic cell tumor/sarcoma (FDCT/S). The association of iT-LBP and FDCT/S contributes to the development of paraneoplastic autoimmune multiorgan syndrome (PAMS), and a dismal prognosis [44].The second most common association is with hepatocellular carcinoma (HCC), followed by acinar cell carcinoma of the parotid. It has also been described together with B- and T-cell lymphomas and autoimmune disorders. Importantly, no transformation or progression to T-LBL has been described.

**Table 2:** Useful criteria for the diagnosis of indolent T-lymphoblastic proliferation (iT-LBP)

TdT+/CD3+ T cells in sheets or dense clusters
Preservation of lymph node architecture
Lack of cellular atypia
No aberrant antigen expression
Cortical T-cell phenotype^§^
Often expression of CD10 and CD99
Lack of LMO2 expression*
CD34 and CD33 mostly negative
Non-clonal TdT+ T cell proliferation
No thymic epithelium present
No bone marrow involvement
No mediastinal involvement

§ most cases are double positive CD4/CD8 but double negative cases have been reported

* LMO2 is positive in most cases of T-acute lymphoblastic leukemia

Data modified from Saglam et al. [10]

**Fig. 4:**
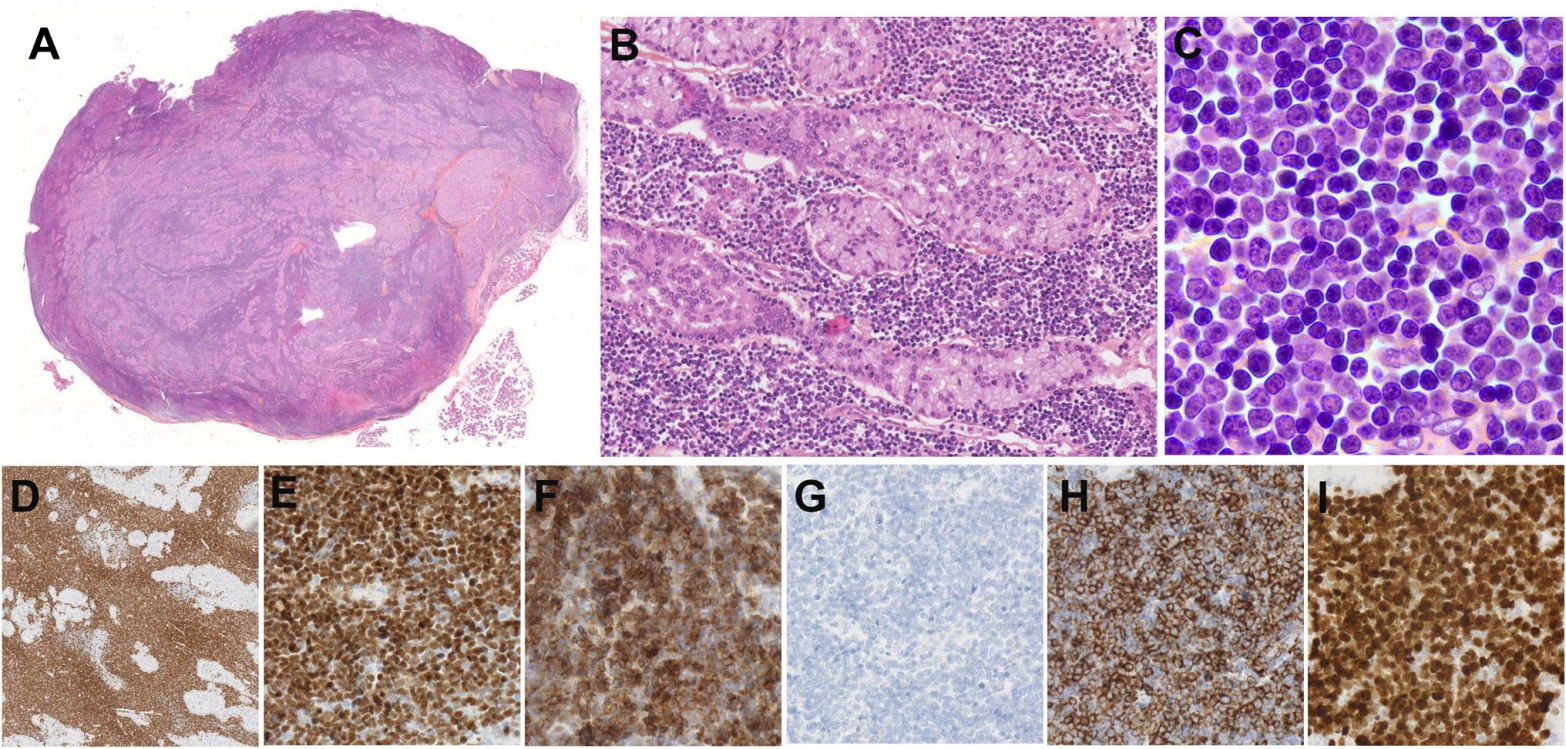
Indolent T-lymphoblastic proliferation (iT-LBP). Case LYWS-246 courtesy of F. Pesce. **A** A panoramic view of the parotid gland showing an epithelial neoplasia accompany of a rich lymphoid infiltrate. Note in the periphery normal parotid gland. **B** Higher magnification showing the neoplastic glandular proliferation consistent with a low-grade acinar adenocarcinoma of the parotid gland surrounded by a small lymphoid infiltrate. **C** The lymphoid infiltrate is composed of small and medium-sized lymphoid cells with round nuclei, open chromatin, inconspicuous nucleolus and scant cytoplasm corresponding to lymphoblasts without atypia. Immunohistochemical analysis revealed that the lymphocytes are positive for **D** CD3, **E** TdT, **F** CD1a, and **H** CD10 and negative for **G** CD34 (G). **I** The Mib1 stain shows a proliferation rate of 100%

Ten cases were submitted to the workshop with the diagnosis of iT-LBP of which the diagnosis was confirmed in 7 cases (Supplemental Table 3). There were 4 men and 3 women with a median age of 40 years (range 35–86 years). Two cases were associated with CD of which one in addition had a FDCT/S, two cases with acinar cell carcinoma of the parotid gland, one each with follicular lymphoma, ovarian cancer metastasis and in tonsils in a patient with clinical history of Langerhans cell histiocytosis. The case presented by F. Pesce (LYWS-246) represented well the morphology and phenotype of the cases submitted (Fig. 4). The patient was an 86-year-old woman with parotid gland enlargement due to carcinoma associated to a dense infiltrate of small, non-atypical lymphoblasts with a cortical phenotype (TdT +, CD3 +, CD4 +, CD8 +, CD1a +, CD10 +, and MIB1 > 95%), and polyclonal TR.

Two cases were submitted with the diagnosis of iT-LBP (LYWS-35 submitted by S. Bunting and LYWS-215 submitted by G.A. Croci). Both cases presented with a mediastinal mass. The diagnosis of iT-LBP in mediastinal masses should be carefully considered due to the presence of residual thymic tissue. Accordingly, the first case represented a neuroendocrine carcinoma (NEC) of the thymus with residual thymus mimicking iT-LBP, and the second case exemplified a true thymic hyperplasia [45]. Case LYWS-196 submitted by Y. Jin was a very difficult case of a 73-year-old woman with generalized lymphadenopathy and relative indolent clinical course diagnosed originally as iT-LBP but in retrospect a diagnosis of T-LBL was rendered. The equivocal TR clonality analysis and the protracted clinical course contributed to the difficult differential diagnosis. The patient died with disseminated disease 5 years after the original diagnosis.

**Fig. 5:**
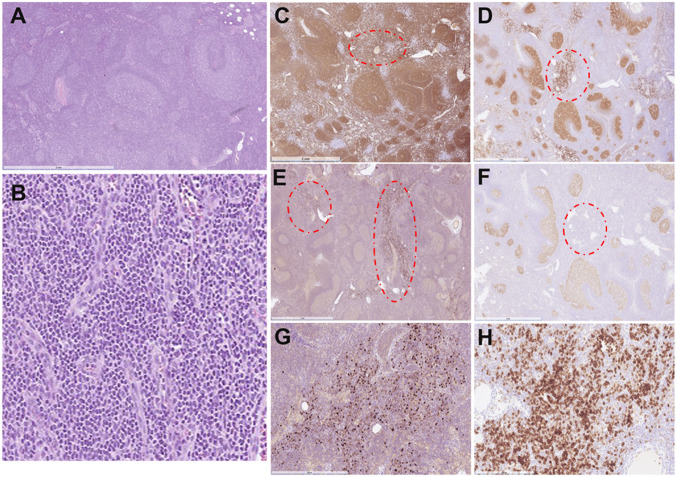
Indolent B-lymphoblastic proliferation (iB-LBP). Case LYWS-373 courtesy of P. Devi. **A** A panoramic view of a lymph node showing follicular hyperplasia. **B** Higher magnification in the interfollicular areas where small lymphocytes, without atypia are observed. **C** CD20 is positive in the hyperplastic germinal centers but also in the interfollicular areas surrounding blood vessels (red circle). **D** CD10 is positive in the germinal center cells but also in the interfollicular lymphoid cells surrounding the blood vessels (red circle). **E** TdT reveals group of positive cells surrounding blood vessels in the interfollicular areas. Note that these cells are CD20 +, CD10 + and TdT + but are negative for **F** BCL6. **G** Higher magnification with TdT stain to demonstrate the numerous TdT-positive cells in the interfollicular areas. **H** CD10 higher magnification from **D** reveals numerous CD10 + cells in the interfollicular area

In contrast to nodal expansion of T-cell precursors that are well described, similar nodal expansions of B-cell precursors (hematogones) are rare. iB-LBP are non-clonal B-cells, CD10 +/TdT +, with only few cases reported [46]. Case LYWS-373 submitted by P. Devi was a unique example of iB-LBP in several LNs in a 48-year-old woman, completely asymptomatic (Fig. 5).

## Other indolent, clonal T-cell lymphoproliferative disorders

Seven cases were submitted to the workshop representing various clonal indolent T-cell lymphoproliferations (Supplemental Table 4). These included 3 cases of transient, clonal CD8 + T-cell proliferations, 2 cases of lymphocyte-variant of hypereosinophilic syndrome (LV-HES), and two cases of PD1 + clonal T-cell LPDs in the skin.

### Transient, clonal CD8 + T-cell expansion

**Fig. 6:**
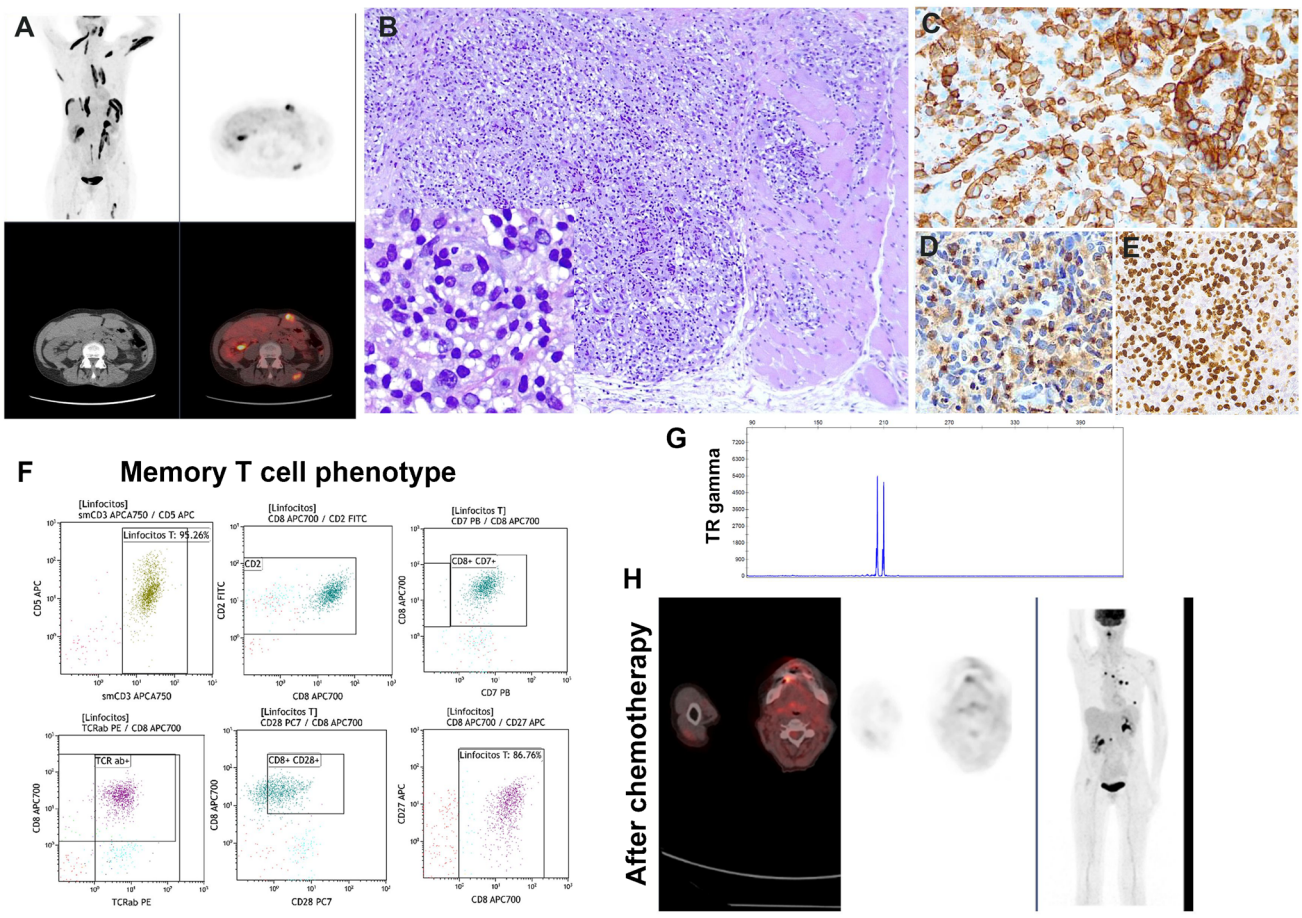
Transient, clonal CD8 + T-cell expansion in a 62-year-old woman with poorly differentiated squamous cell carcinoma of the oropharynx. Case LYWS-344 submitted by M. Gomez-Tena. **A** 18F-PET/CT analysis demonstrating multifocal 18F- deposition suggestive of metastatic disease. **B** Muscle biopsy showing an atypical lymphoid infiltrate. Insert: the lymphoid infiltrate is composed of medium to large-sized cells with irregular nuclei and broad clear cytoplasm. The atypical cells are **C** CD8-positive, **D** express TIA1, and show a high proliferation rate with **E** MIB1 stain. **F** Flow cytometry reveals a memory T-cell phenotype with expression of CD28 and CD27. Note that the CD8 positive cells show normal expression of CD5, CD2, and CD7. The cells have a TCR alfa-beta phenotype. **G** TR gamma demonstrates a bi-allelic monoclonal peak. **H** 18F-PET/CT analysis demonstrates almost complete resolution of the muscle lesions after only one dose of cisplatin

Transient clonal, self-limited, CD8 + T-cell expansions usually are the result of a very restrictive immune-mediated cytotoxic T-cell response to virus or neoantigens that always create uncertainty in the diagnosis. Aside from the transient clinical behavior, there are no objective criteria to differentiate transient, clonal CD8 + T-cell expansions from peripheral T-cell lymphomas (PTCL), which is the most important pitfall. The three cases submitted represented clonal CD8 + T-cell expansions in different clinical settings including infectious mononucleosis (IM) (LYWS-330 submitted by L. Quintanilla-Martinez), autoimmune disease (LYWS-276 submitted by M. Pizzi), and carcinoma (LYWS-344 submitted by M. Gomez-Tena). Case LYWS-344 was a striking case of a 62-year-old woman that was diagnosed with a squamous cell carcinoma of oropharynx, human papilloma virus (HPV), and EBV negative. PET/CT demonstrated multiple hypermetabolic deposits in muscle suggestive of metastasis (Fig. 6). However, a muscle biopsy revealed a clonal CD8 + T-cell infiltrate that by flow corresponded to CD8 + memory cells (CD27 +, CD28 ±). Clonality analysis revealed identical TR clones in all samples. NGS analysis was negative for mutations. A diagnosis of PTCL, not otherwise specified (NOS), was considered. The patient received treatment for the carcinoma and after a single dose of cisplatin the lymphoid infiltrate disappeared completely. This case is very instructive disclosing that the clinical context, despite the scaring looking lymphoid infiltrate, and understanding that clonal proliferation does not necessarily mean malignancy, are essential to avoid the wrong interpretation of PTCL, NOS. The unusual accumulation of clonal CD8 + T-cells in muscles most probably reflected an ongoing T-cell response towards circulating tumor antigens. Different grades of CD8 + memory T-cells with restricted TR repertoire have been reported in several malignancies [47–49]. Case LYWS-330 was a 34-year-old young man that presented with spontaneous splenic rupture. The spleen and the liver were infiltrated by a clonal CD8 + T-cell infiltrate that raised the diagnosis of hepatosplenic T-cell lymphoma. The EBER in situ hybridization showed numerous positive cells and double-stains revealed that the EBV positive cells were CD20 + B-cells. This case raised two important issues; first, the need to perform EBV stains in cases of spontaneous spleen rupture since up to 15% of all splenic ruptures are attributable to EBV infection, and second, the importance of performing double-stains to define the phenotype (B vs T) of the EBV + population, which is necessary to establish an accurate diagnosis. Identification of clonal CD8 + T-cell population in IM is not unusual. In a recent series, 4/6 cases of splenic spontaneous rupture secondary to IM, revealed a clonal CD8 + T-cell population, and like in this case, all patients recovered completely [50]. The third case (LYWS-276) was a 21-year-old young man with celiac disease that presented with meningitis. Flow cytometry in spinal fluid detected 72% of abnormal T-cells. The same cells were detected in PB and BM with identical clonal TR. HHV7 infection was demonstrated and the patient was treated with steroids and anti-viral therapy. The patient recovered completely and is asymptomatic 32 months after this event. In all three patients the diagnosis of PTCL and NOS was entertained. It is important to be aware of the existence of transient, clonal CD8 + T-cell proliferations to avoid the pitfall of PTCL, NOS and treat the patient unnecessarily.

### Lymphocyte variant of hypereosinophilic syndrome (LV-HES)

LV-HES is considered a reactive condition characterized by the presence of an abnormal, clonal, Th2 T-cell population with abnormal production of IL-5 and elevated IgE in serum resulting in HES and cutaneous manifestations (81%), infiltration in LNs (62%), joints (29%), GIT (24%), and other organs [51, 52]. Flow cytometry reveals the characteristic abnormal phenotype in PB with lack of surface CD3 expression (sCD3-), CD5^bright^, CD4 + and often loss of CD7 expression [53].The sCD3- and CD4 + phenotype detected by flow cytometry is not present in healthy individuals and should alert the pathologist to include LV-HES in the differential diagnosis. In contrast to flow cytometry, the T-cell infiltrate in tissues is CD3 +, because cytoplasmic CD3 (cCD3) is preserved. This disorder has very good prognosis with excellent response to steroids or anti-IL-5 therapy. The differential diagnosis with PTCL is not always easy, especially with TFH lymphomas and Sézary syndrome (SS). TFH markers are always negative and help in the differential diagnosis. *STAT3* variants are the only variants detected so far, although infrequently [53]. The two cases of LV-HES submitted to the workshop (LYWS-380 submitted by A. Kwiecinska and LYWS-212 submitted by D.I. Laczko), demonstrated the clinicopathological features of the disease. Both cases were very well documented with atypical T-cells in PB, BM, and/or skin. In case LYWS-380, a *STAT3* c1852G > C p.Gly618ATArg variant was identified supporting the contention that *STAT3* activation might be important in the pathogenesis of LV-HES [54].

### PD1 + clonal T-cell proliferations in the skin

Two very difficult cases were submitted with the diagnosis of PD1 + T-cell proliferations of the skin with indolent behavior. Case LYWS-408 submitted by L. Colomo was a 52-year-old man that presented with pruritic lesions involving trunk and chest, neck, axillary, and inguinal areas. The lesions were recurrent and persistent despite steroid treatment. Several skin biopsies in a period of 4 years were available. All skin biopsies looked the same and had the same phenotype and identical clonal TR rearrangement. The phenotype was CD3 +, CD4 +, CD5 +, PD1 +, CXCL13 +, BCL6 +, CD10 +, CD7- and low proliferation. Flow cytometry did not demonstrate circulating atypical cells. The case was difficult to classify; however, the panel agreed with the submitter that the case represented an indolent clonal TFH, atypical cutaneous proliferation without systemic involvement after 4 years. Case LYWS-449 submitted by G. Rassidakis was a 76-year-old female with 5-year clinical history of extensive erythroderma and an atypical T-cell infiltrate demonstrated by flow cytometry in PB, skin, and LNs; CD3 +, CD4 +, CD5 +, PD1 +, CD7-, CD26 +, CD2^dim^, and TCRb1 + and in skin a subset was CD30 +. The same clonal TR rearrangement was demonstrated in all specimens and 3 pathogenic variants in *ARID5B*, *CHD1*, and *FAT1* were detected with NGS. In both cases, the diagnosis of SS was discussed. SS is defined by the triad of erythroderma, generalized lymphadenopathy and clonal T-cells with cerebriform nuclei. The phenotype of the infiltrating cells is usually CD3 +, CD4 +, and CD8- with variable loss of CD7, CD26, and CD2 expression. PD1 expression is a typical finding in SS. Furthermore, the expression of PD1 together with lack of CD7 expression supports the diagnosis of SS in patients with erythrodermia [55, 56]. Despite the expression of CD26, the panel favored in the latter case the diagnosis of SS.

## EBV-positive lymphoproliferative disorders

Six cases were submitted with the diagnosis of “indolent” EBV + LPDS (Supplemental Table 5). Two cases were examples of extranodal NK/T-cell lymphoma, nasal type with long follow-up (LYWS-340 submitted by T. Pikivaca and LYWS-293 submitted by L. Bonngiovanni). The case LYWS-293 had a *RIPK1* germline mutation. Two cases represented EBV + polymorphic LPDs in HIV setting (LYWS-61 submitted by D. Even Fridrich and LYWS-138 submitted by T. Lakic). One case was a CAEBV disease that progressed to a CD8 + PTCL (LYWS-250 submitted by P. Devi). The last case (LYWS-363 submitted by A. Toklu) was a diffuse large B-cell lymphoma with chronic inflammation. Although initially these cases might have had a protracted clinical presentation, most cases were treated with chemotherapy and some patients even died of disease. The panel agreed that these cases do not represent and should not be considered as indolent LPDs.

## Intestinal lymphoproliferative disorders

Intestinal LPDs are a group of indolent B-, T-, or NK-cell LPDs involving primarily the GIT without systemic manifestations. In the workshop, 14 cases were submitted with the diagnosis of indolent clonal T/NK-cell LPD of the GIT (iT/NK-LPD-GI), 6 cases of low-grade B-cell lymphoma including 5 cases of DFL, and one case of MALT lymphoma involving the duodenum.

### Indolent clonal T/NK-cell lymphoproliferative disorders of the GI

iT-LPD-GI is a rare clonal, non-destructive and non-epitheliotropic T-cell LPD occurring in adults with a male predominance. It involves any site in the GIT, most commonly the small bowel and colon [3, 57]. Since its first description in 1994 [58], approximately 80 cases have been reported in the literature. The disease appears more heterogeneous than previously believed in terms of histology and outcome [57]. The cells usually express pan-T-cell markers with variable loss of CD5 and CD7. TIA1 is positive; however, Granzyme B and perforin are negative. The cells show a TCRβF1 phenotype with inconsistent expression of T-bet and GATA3. TFH markers, CD30, CD56, and EBER are negative. CD20 and CD103 may be expressed [59, 60]. Proliferation is very low (< 5%), and by definition the disease is TR monoclonal. Genetic alterations in the JAK/STAT pathway include *STAT3::JAK2* fusion, *STAT3* variants, or *SOCS1* deletion but no *SETD2* alterations have been identified [59–61]. Recurrent alterations in genes involved in NF-kB signaling or epigenetic modifiers, as well as structural alterations of IL2 especially in CD8 + cases, have been reported [60]. Clinically, the majority of patients have an indolent course but some rare cases may progress or even transform [3, 57]. Because of the rare cases reported to have transformed, the 5th edition of the WHO lymphoma classification decided to adopt the name of “indolent T-cell lymphoma of the GI tract.” However, cases that have transformed seem to have different clinicopathological features (see below).

In the workshop, 14 cases were submitted with the initial diagnosis of iT/NK-LPD-GI (Supplemental Table 6). In 13 cases the panel agreed with the diagnosis. According to clinicopathological features, 3 groups were recognized: (1) iT/NK-LPD-GI with typical features (*n* = 6); (2) iTLPD-GI with unusual features (*n* = 4); and (3) “other” intestinal T-cell LPD/lymphoma (*n* = 3). The remaining case concerned a 37-year-old patient with Crohn’s disease and oligoclonal T-cell expansion in the intestinal mucosa (LYWS-191 submitted by M. Moore). The panel favored the diagnosis of reactive infiltrate secondary to Crohn’s disease. The case demonstrated the difficult differential diagnosis and sometimes overlapping features with IBD.

### iT/NK-LPD-GI with typical features

The 6 cases submitted illustrated the clinical and morphological features described in the disease (Table 3). Five cases were of T-cell phenotype and one of NK-cell phenotype. The median age at presentation was 42 years (range 16–67 years) with a male to female (M:F) ratio of 1:1. All patients had digestive symptoms with endoscopic lesions involving the small bowel. Histologically, iT-LPD-GI typically showed small to intermediate-sized lymphocytes infiltrating the lamina propria without destroying the glands and without epitheliotropism. Three cases had a CD8 +/CD4- phenotype (LYWS-211 submitted by N. Vidal-Robau, LYWS-349 submitted by E. Hookway and LYWS-454 submitted by A. Green), and two were CD8/CD4-negative (LYWS-24 submitted by J. Karrs and LYWS-328, submitted by D. Nann). All cases revealed low proliferation (~ 5%). Only one case expressed PD1 (1/4) and two cases showed aberrant CD20 expression. All cases were monoclonal. NGS was performed in 3 cases. Non-recurrent gene alterations were identified in two cases including a *FAT1* variant (Case LYWS-24), and a heterozygous *IRF8* splice region variant (LYWS-349), co-occurrent with a *STAT5b* missense variant of uncertain significance. As described in the literature [57], the cases with available follow-up had a stable disease without treatment or were in remission under steroid therapy or other immune suppressive treatment.

**Table 3:** Comparison of clinicopathological features of 13 cases with the diagnosis of indolent T/NK-cell lymphoproliferative disorder of the gastrointestinal tract (iT/NK-LPD-GI)

Clinicopathological features	iT/NK-LPD-GI with typical features (6 cases)	iT-LPD-GI with unusual features (4 cases)	Other intestinal T-LPD/lymphoma (3 cases)
Age	41 years (range 16–67 years)	66 years (range 37–74 years)	42 years (range 39–67 years)
Sex	M:F = 1:1	M:F = 3:1	M:F = 3:0
Sites of involvement	Small bowel (LYWS-24; LYWS-211; LYWS-328; LYWS-349; LYWS-454; LYWS-79)	Small bowel (LYWS-461*; LYWS-253; LYWS-256; LYWS-96)	- Gastric (LYWS-337) - Gastroduodenal (LYWS-364*) - Epiglottis (LYWS-376)
Extension/dissemination	No extension/dissemination reported	- Wall layers extension (3/3) - Mesenteric LN (2/4) - Splenomegaly (1/4)	- Mesenteric LN (1/3) - splenomegaly (1/3) - PB/BM (3/3) - Remote LN (2/3) - Skin (1/3)
Immunophenotype	CD8 +/CD4- (3 cases) CD8-/CD4 + (2 cases CD56 +/CD8^dim^ (1 case) - Loss or weak CD5 and CD7 (2 cases, each)	CD4 +/CD8- (2 cases) CD8 +/CD4- (2 cases) - Loss or weak CD5 and CD7 (1 case, each)	CD4 +/CD8- (3 cases) - No loss of CD5 and CD7
Weak CD20 expression	2/3 cases	1/4 cases	None
PD1 expression	1/4 cases	0/4 cases	3/3 cases
Proliferation index	< 5%	< 5%	> 10%
Clonality analysis	Monoclonal TR (5 cases) NK-phenotype polyclonal	Monoclonal TR (3 cases)	Monoclonal TR (3 cases)
Mutational profile	- *FAT1* mutation (LYWS-24) - *IRF8* splice region variant and *STAT5b* missense VUS (LYWS-349) -*JAK3* mutation (LYWS-79)	*- DNMT3A* mutation (LYWS-461*)	*- STAT5* mutation (LYWS-337) *- JAK1* and *TET* VUS mutations (LYWS-364*)
Outcome	- Stable disease (5 cases) - NK-phenotype LFU	- Stable disease (3 cases) - LYWS-256 LFU	- Stable disease (2 cases) - 1 case transformed to PTCL, NOS

*BM* bone marrow, *LYWS* lymphoma workshop, *LN* Lymph node, *PB* peripheral blood, *TR* T-cell receptor genes, *VUS* mutation of uncertain significance, *LFU* lost to follow-up

^*^Case presented in the workshop

iNK-LPD-GI is a very rare disease with only few cases described, and recently recognized in the ICC/WHO classifications [1, 2, 62]. In contrast to iT-LPD-GI, iNK-LPD-GI tends to affect young adults, show more frequent cellular atypia, and *JAK3* variants have been reported in 30% of cases [63, 64]. Accordingly, case LYWS-79 submitted by M. Shi corresponded to a 35-year-old woman with disease in the small bowel, NK-cell phenotype, and two *JAK3* variants. In this case, a diagnosis of EBV-negative NK-cell lymphoma was considered, a potential pitfall. The iNK-LPD-GI was complicated by Crohn’s disease, an association that has been reported with iT-LPD-GI [3, 65]. The differential diagnosis with Crohn’s disease is not always easy (LYWS-191). The demonstration of monoclonality or the presence of gene alterations (i.e., *JAK3*) favors the diagnosis of iT/NK-LPD-GI.

### iT-LPD-GI with unusual features

**Fig. 7:**
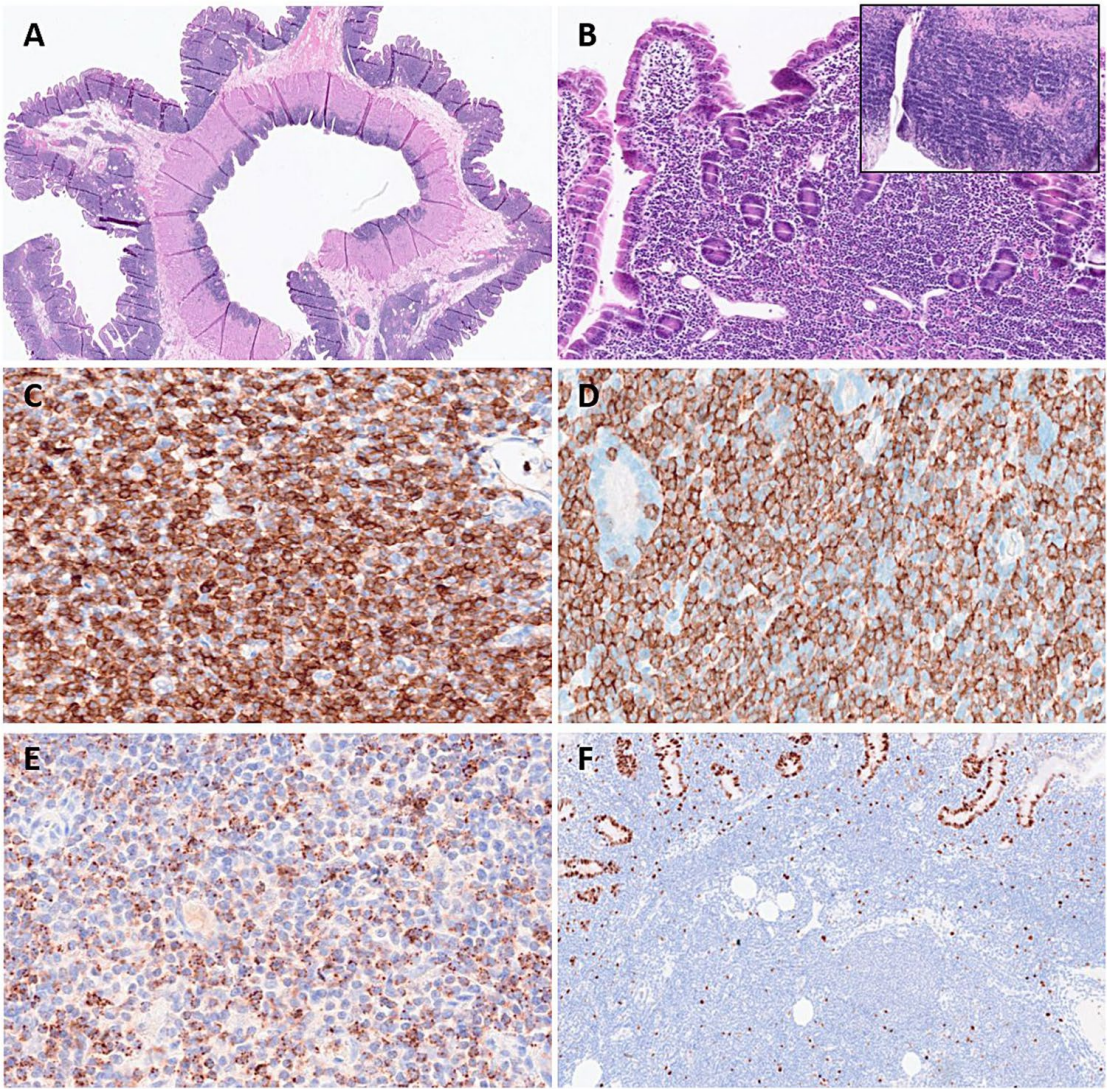
Indolent clonal T/NK-cell lymphoproliferative disorder of the GI with unusual features. Case LYWS-461 courtesy of P. Gaulard. **A** Panoramic view of the duodenal wall showed diffuse band-like lymphoid infiltrates in the whole mucosa and serosa, sparing the muscular layers. **B** Higher magnification showed monotonous small- to medium-sized lymphoid cells in the lamina propria that spread to the serosa (insert). The lymphoid infiltrate is positive for **C** CD3, **D** CD8, and **E** TiA1.** F** Ki67 proliferative index was < 5%

This second group had similar clinical presentation as the previous group, but morphologically, the T-cell infiltrate extended to all intestinal wall layers and in some cases mesenteric LNs were involved (Table 3). The median age was 66 years (range 37–74 years) with M:F ratio of 3:1. The case LYWS-461 presented by P. Gaulard was a unique case that showed a band-like T-cell infiltrate in the duodenal mucosae and in the serosa, sparing the muscular layers (Fig. 7). The other three cases (LYWS-253 submitted by L. Goh; LYWS-256 submitted by F. Climent and LYWS-96 submitted by C. Abou-Seif) had infiltrates that diffusely extended to all layers of the jejunal wall and in some cases to mesenteric LNs and/or splenomegaly. Two cases were CD4 +/CD8-, and two were CD8 +/CD4- or partially positive. PD1 was negative, and proliferation rate was low (~ 5%). Case LYWS-256 had a CD56 + lymphoid subpopulation, a rare but well-described finding. The three cases with follow-up in this subgroup showed no progression.

### “Other” intestinal T-cell LPD/lymphoma

**Fig. 8:**
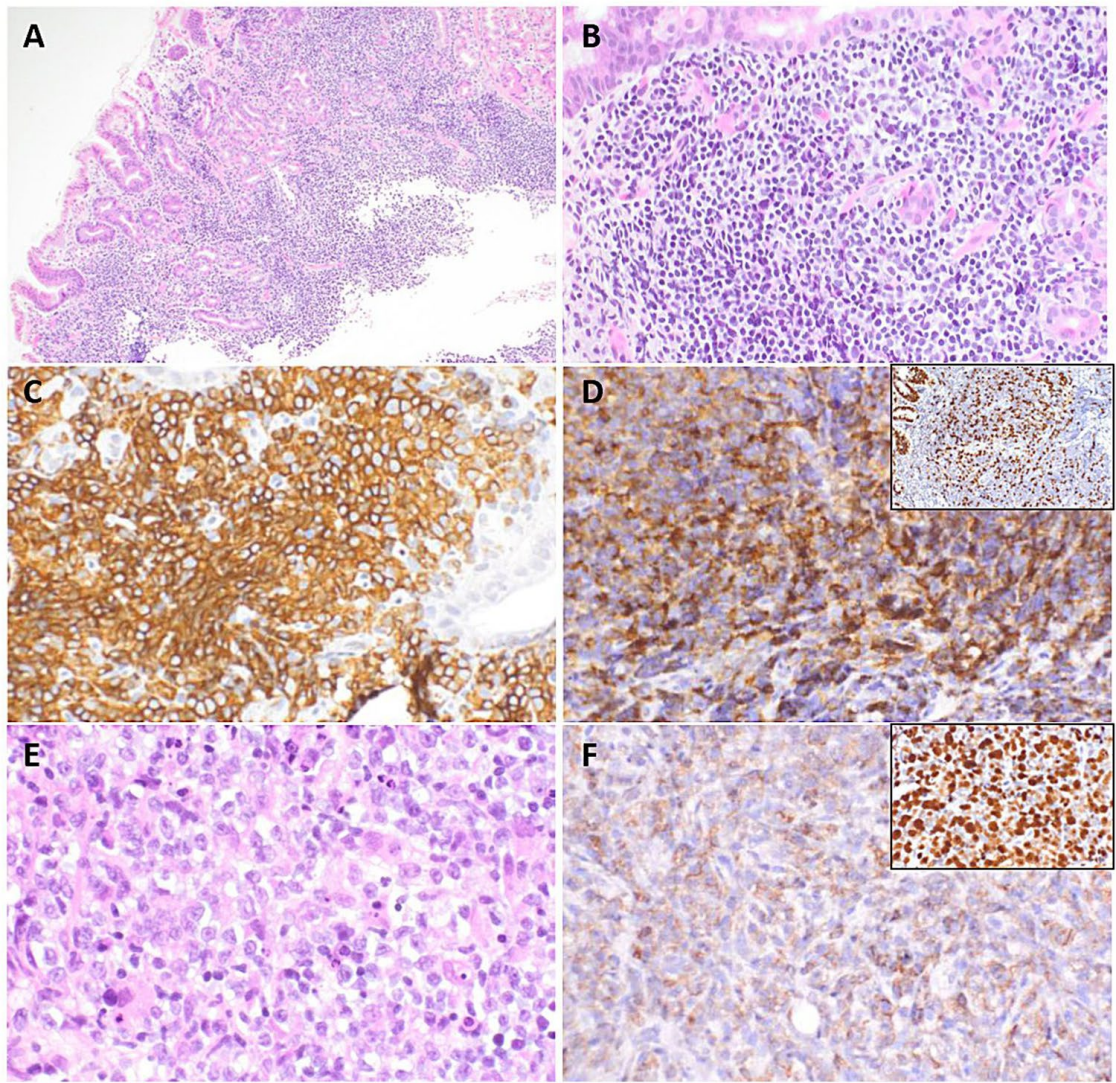
Indolent, systemic PD1 positive T-cell lymphoma/lymphoproliferative disorder with intestinal tropism. Case LYWS-364 courtesy of E. M. Gerhard-Hartmann. **A, B** Gastro-intestinal biopsy showed a dense infiltrate of small- to medium-sized lymphocytes with round or mildly irregular nuclei in the lamina propria with extension into the submucosa. The lymphoid infiltrate was (**C**) CD3 and (**D**) PD1 positive, with Ki67 index > 20% (insert). Biopsy from a sacral lesion showed progression to (**E**) an aggressive T-cell lymphoma with (**F**) large atypical lymphoid cells expressing PD1 with high Ki67 index (insert)

This group included three cases with intestinal involvement but disseminated disease clearly separating these cases from the previous two subgroups (Table 3). The case LYWS-364 presented by E. M. Gerhard-Hartmann corresponded to a 39-year-old man with initial diagnosis of iT-LPD-GI associated with retroperitoneal lymphadenopathy and splenomegaly. The infiltrate had a CD4 +/CD8- phenotype and expressed CD20 and PD1 with proliferation rate > 20% (Fig. 8A–D). The patient was in W&W strategy but progressed 3 years later with PB and BM involvement. After a good response to anti-CD52 treatment, he relapsed a year later with a more aggressive T-cell lymphoma infiltrating LNs, lungs, orbit and sacrum and was treated with chemotherapy (Fig. 8E, F). The second case LYWS-376 submitted by P. Cervera, concerned a 67-year-old man suffering from a swollen epiglottis with past-history of vasculitis and lymphadenopathy for 10 years. The epiglottal biopsy revealed a CD4 +/PD1 + small T-cell infiltrate with identical monoclonal TR in the skin and LN. Of note, this patient did not have GI lesions confirmed by endoscopic examination. The last case LYWS-337 submitted by L Mescam corresponded to a 40-year-old man with abdominal symptoms. A mesenteric LN biopsy showed a clonal, low-grade CD4 +/PD1 + T-cell LPD with a *STAT3* variant. During the staging procedures, the same T-cell clone was identified in PB and BM. Endoscopic examination revealed the same clonal T-cell infiltrates in serial intestinal biopsies with identical *STAT3* variant. The last two cases showed a relative indolent clinical presentation with no progression.

Altogether, iT-LPD-GI is a clonal T-cell LPD with low-grade morphological features often limited to the intestinal mucosa but sometimes can spread to all wall layers or be associated with mesenteric LN involvement. Dissemination or progression to a more aggressive disease is rare. Nevertheless, three cases were submitted to the workshop that did not conform to iT-LPD-GI as typically defined. These cases, in addition to GI involvement, had disseminated disease to PB, BM, LN and/or skin at presentation with an indolent clinical course. Moreover, these cases were CD4 +/PD1 + with proliferation rates > 10%. Remarkably, cases reported in the literature of putative iT-LPD-GI with dissemination or progression were either CD4 + or double CD4/CD8-negative [3]. However, the cases were not investigated for PD1 expression. Nevertheless, in a large series of iT-LPD-GI, it was demonstrated that PD1 is not expressed. Interestingly, a more recent series identified 2/10 cases with PD1 expression, one was CD4 + and the second double CD4/CD8-, indicating that lesions with these two phenotypes have a higher probability to express PD1 and disseminate [60]. Accordingly, the only case of iT-LPD-GI in the workshop that showed weak staining for PD1 (LYWS-24; 1/7 cases), was a case double CD4/CD8-negative and carried a *FAT1* variant, a gene alteration that has been identified in PTCL and linked with worse prognosis [66]. The expression of PD1 seems to identify cases with potential for a more disseminated disease. The panel believed that these PD1 + disseminated cases do not belong to the iT-LPD-GI spectrum and most probably represent a different entity with intestinal tropism. The provisional name of “indolent, systemic, PD1 + T-cell lymphoma/LPD” was proposed for this group of cases to emphasize the phenotype and the indolent clinical course, despite systemic dissemination.

### Duodenal-type follicular lymphoma

DFL has well-defined criteria in both WHO/ICC classifications [1, 2, 67]. DFL is considered an early FL lesion, occurring in adults, most often in female and predominantly found in the second portion of the duodenum. Histologically, it resembles FL grades 1–2. Phenotype and molecular profiles are similar to FL but with low frequencies of mutations and less genomic complexity [68, 69]. Most patients are conservatively managed [70].

In the workshop, 6 cases were submitted with the diagnosis of DFL (Supplemental Table 7). In five cases the diagnosed was confirmed by the panel. The median age was 53 years (range 36–65 years) with M:F ratio of 4:2. Five cases represented typical examples of DFL. The case LYWS-396 submitted by U. Sakhadeo concerned a 44-year-old woman presenting with a diffuse proliferation of small B-cells with plasma cell differentiation, in the duodenum. Absence of GC markers, lack of *BCL2*-rearrangement (R) by FISH, and the presence of a *CD79b* variant without canonical FL mutations were more consistent with a MALT lymphoma infiltrating duodenum.

## Other indolent B-cell lymphoma/lymphoproliferative disorders

The 11 cases of B-cell LPD/lymphomas with low-grade or indolent features included in this group were heterogeneous and separated in 3 subgroups: (1) indolent, extranodal FL/FL-related lesions (*n* = 3); (2) isolated atypical follicle with genomic alteration (*n* = 2); and (3) single examples of well-defined low-grade B-cell lymphomas (*n* = 6) (Supplemental Table 8).

### Indolent extranodal FL/FL-related lesions

**Fig. 9:**
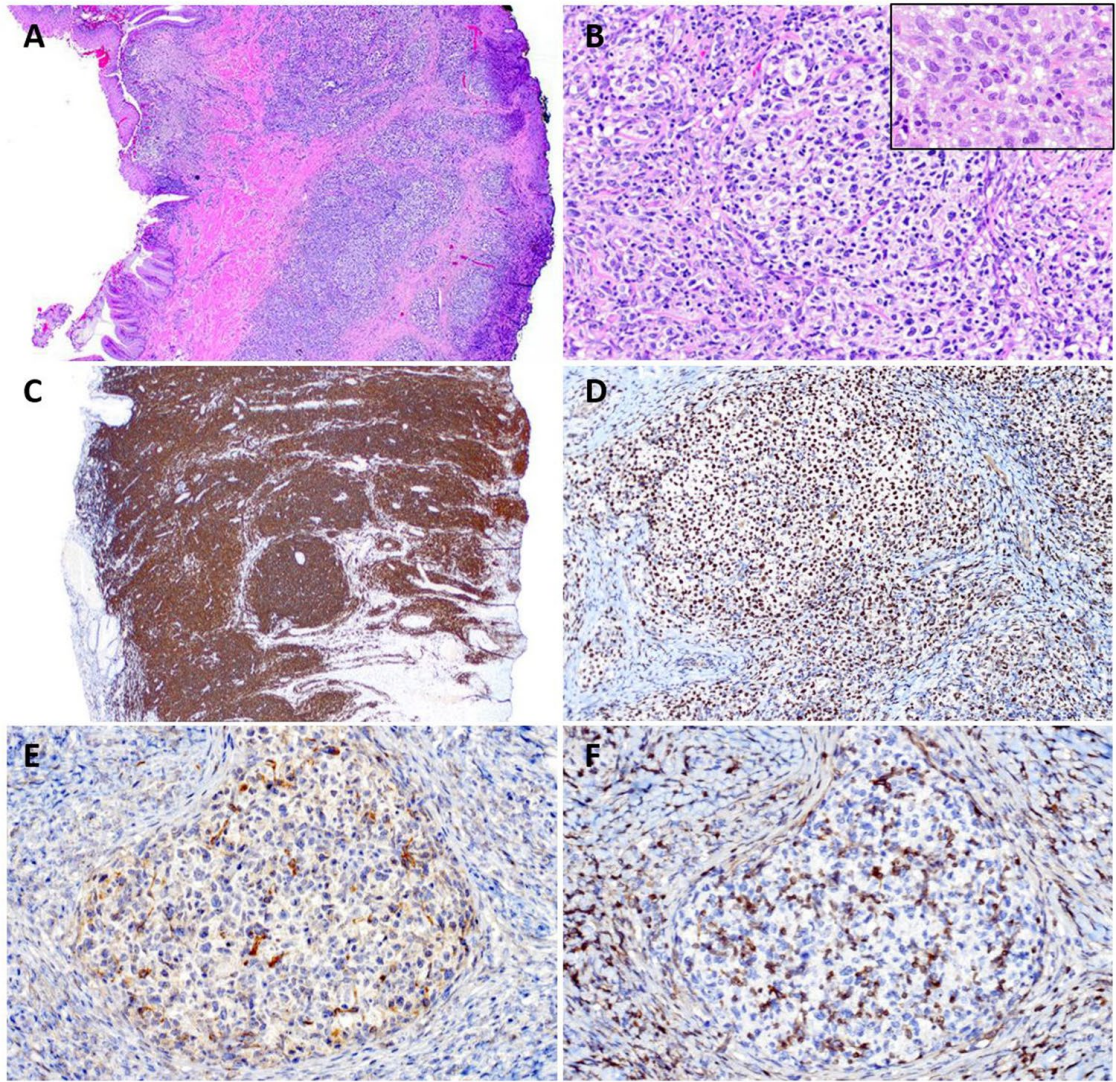
Follicle center lymphoma of the lower female genital tract. Case LYWS-317 courtesy of V. Leventaki. **A** Panoramic view of uterine cervix showed a submucosal lymphoid infiltrate with follicular and focally diffuse pattern. **B** Higher magnification showed a predominance of large centrocytes and occasional centroblasts. Tumor cells were **C** CD20 positive, **D** BCL6 positive, and **E** CD10 negative. **F** BCL2 was negative

Three cases of FL or FL related lesions were received. The first case LYWS-317 presented by V. Levantaki corresponded to a 22-year-old female with persistent abnormal uterine mass without LN involvement. Cervical-uterine biopsy was diagnostic of a follicle center lymphoma (FLC) of the lower female genital tract. This rare variant of FL has been recently characterized [4]. It is a localized disease in the cervix and vagina without evidence of systemic involvement. The tumor is composed of predominant large centrocytes with occasional centroblasts infiltrating the subepithelial mucosa with follicular and diffuse pattern and without surface erosions or ulceration (Fig. 9). Similar to cutaneous follicle center lymphoma (PCFCL), tumor cells express BCL6 but are CD10 and BCL2 negative, lack *BCL2*-R, and carry frequent alteration of *TNFRSF14* (60%) without *CREBBP* and *KMT2D* mutations [71]. Accordingly, in case LYWS-317, *TNFRSF14*, *S1PR2*, *IGLL5*, and *EZH2* variants were detected. Despite often containing a significant proportion of large B-cells, FCL of the lower female genital tract has a good outcome, with durable clinical response following treatment, low risk of recurrence and virtually no risk of systemic dissemination.

Case LYWS-338 submitted by M. Recuero Pradillo corresponded to a FL with *MYC*-R and *BCL2*-R (“double-hit”). This case concerned a 45-year-old woman with a “double-hit” FL involving the oral cavity. She was treated with surgical excision only with no progression or recurrence after 2 years of follow-up. A total of 49 cases of “double-hit” FL have been reported [72–74]. Most of them had a nodal localization, grade 1/2 cytology, and advanced-stage disease (III-IV) [74]. Importantly, only 17% of cases (*n* = 6/36) transformed into aggressive lymphoma and four of these died of lymphoma [74]. Based on this limited available data, FL-*BCL2*-R/*MYC*-R should not be included within the category of high-grade B-cell lymphoma since most of the cases have a good outcome [1, 67] At this point, it is not recommended to perform *MYC* FISH analysis for FL routine diagnosis.

### Isolated atypical follicle with genomic alteration

**Fig. 10:**
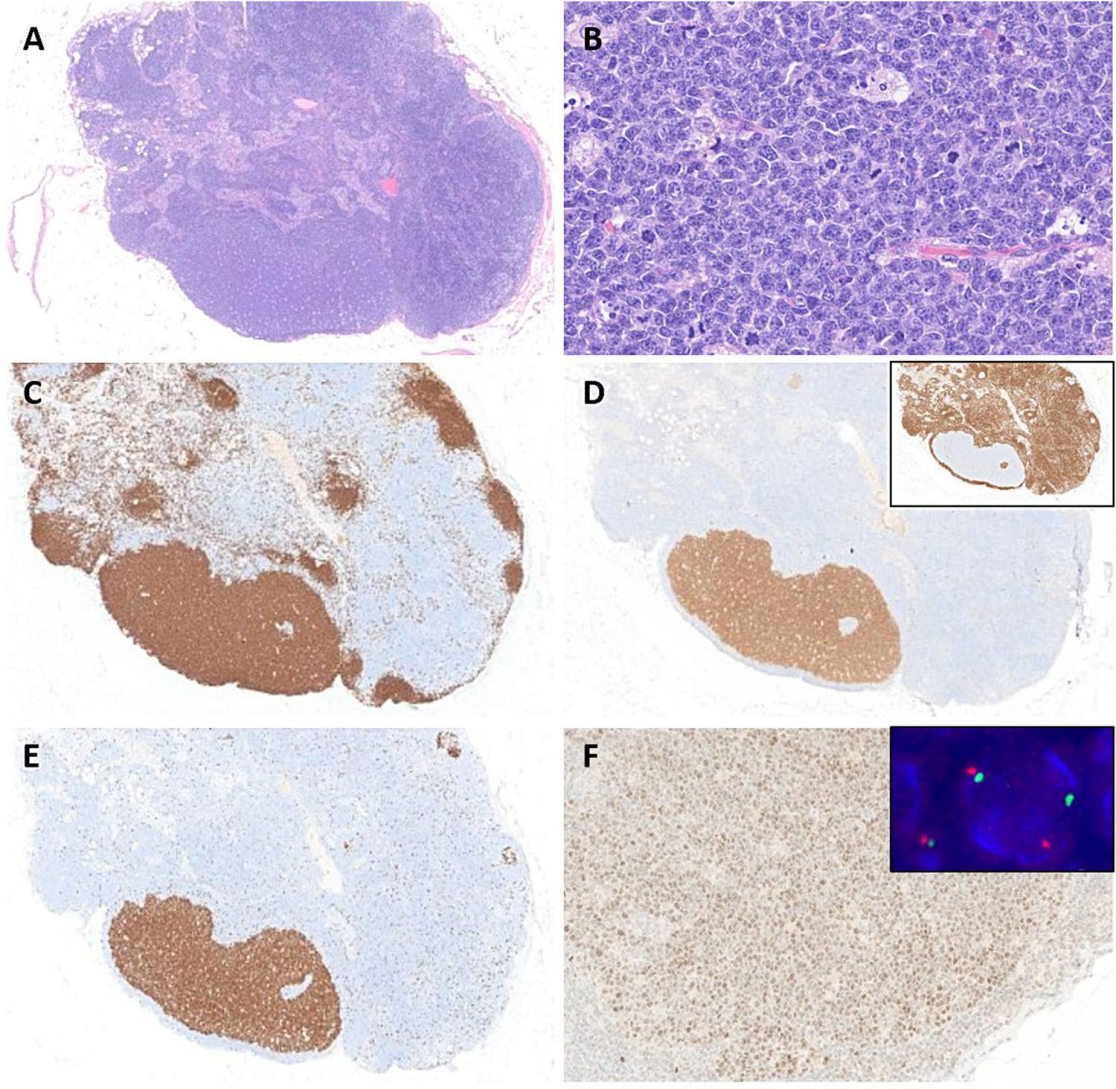
Incipient Burkitt Lymphoma. Case LYWS-38 courtesy of M. Yabe. **A** Panoramic view of a lymph node biopsy showing one single atypical follicle in a reactive lymph node.** B** Higher magnification of the atypical follicle demonstrates a predominance of medium-sized blasts. Atypical follicle is **C** CD20 and **D** CD10 positive, and BCL2 negative (insert). **E** Atypical follicle with loss of polarization showing high and diffuse Ki67 stain. **F** Atypical follicle is MYC protein positive and harbored a *MYC* rearrangement (*MYC*-R) using *MYC* break-apart probes (insert) without *BCL2*-R and *BCL6*-R (not shown)

Rarely, cases with isolated follicles with genetic alterations have been reported. These usually represent incidental findings that cause uncertainty in the diagnosis. Case LYWS-38 presented by M. Yabe corresponded to a 46-year-old woman with a sentinel LN excision for breast carcinoma in situ. LN contained one single atypical follicle with Burkitt lymphoma morphology and harboring *IGH::MYC* fusion consistent with the diagnosis of incipient Burkitt Lymphoma (Fig. 10). Case LYWS-319 submitted by J. Sidhu corresponded to a 17-year-old young man with an isolated LN showing one atypical follicle rich in centroblasts and with *BCL6* polysomy. Both patients were disease-free 18 months and 5 years after LN surgical excision, respectively. To date, only 4 cases of *BCL2*-R negative centroblast-rich isolated atypical follicles and 2 cases of in situ/incipient Burkitt lymphoma have been reported in the literature [75, 76]. Importantly, reported patients with isolated atypical follicles had no overt lymphoma and are disease-free after surgery only.

### Single examples of well-defined low-grade B-cell lymphomas/LPD

Six cases submitted to the workshop represented isolated examples of well-defined low-grade B-cell lymphomas (Supplemental Table 8). Although these cases were not aggressive, they are not really considered within the group of indolent LPDs because all were treated with chemotherapy. Nevertheless, there were two interesting cases submitted by E. F. Mason (LYWS-358) and I. Blázquez Muñoz (LYWS-407). These two cases represented unusual extranodal presentations of SOX11 negative, non-nodal MCL; one case occurred in the stomach (LYWS-358), and one with ocular involvement (LYWS-407). The latter case showed plasma cell differentiation and a non-canonical *MYD88* S219C variant. Finally, case LYWS-419 submitted by L. Carrillo was diagnostic of a cold agglutinin disease with marked erythrophagocytosis in 82-year-old woman.

## Conclusions

Although there are currently no consensus criteria on what defines an indolent LPD, the cases submitted to the workshop revealed certain clinicopathological characteristics that may help to establish some criteria (Box 1). Importantly, the presence of genetic alterations and/or clonal lymphoid proliferations are not enough to consider a lymphoid proliferation malignant. Awareness of the existence of iL-LPDs aims to reduce patient stress and prevent unnecessary treatment. The clinicopathological context is important, as demonstrated by cases with transient, clonal CD8 + T-cell LPD, iT-LPD-GI, or the primary cutaneous clonal B- and T-cell LPDs, previously considered lymphomas. All these lymphoproliferations belong to the group of clonal LPDs with indolent behavior, rare dissemination, with minimal or no treatment. A second group includes the non-clonal LPDs with histologically aggressive features, high proliferative rates but indolent clinical behavior. The example is iT-LBP, where well-established criteria help in the differential diagnosis with T-ALL. A third group is characterized by well-defined lymphomas with indolent behavior despite no treatment. This group includes DFL, some MALT lymphomas and extranodal *BCL2*-R negative FCL. Unusual cases of ENKTCL and TFH lymphomas are also recognized. The reason for the indolent behavior in some aggressive lymphomas is not well understood and warrants further investigation.

**Table:** BOX 1

There is a group of clonal, primary cutaneous LPDs with indolent behavior, which includes PC-MZLPD, PC-CD4+T-cell LPD and PC-CD8+T-cell LPD. These lesions were previously considered lymphomas; however, due to their excellent prognosis the term LPD is preferred.
PC-MZLPD is divided in two morphological subtypes (IGH class-switched and non-class-switched type) that differ in some histological features, IGH expression and microenvironment. Both subtypes have excellent prognosis.
EBV+ PC-MZLPD most often develops in various settings of immune compromise, especially in the posttransplant setting, immunosuppression or immune senescence.
There are overlapping features between PC-MZLPD and PC-CD4+T-cell LPD. Common features include increased numbers of TFH cells, clusters of B cells, plasma cells with light chain restriction and dual IG and TR clonal populations. These are not composite lymphomas.
Transient, clonal CD8+ T-cell LPDs are usually the result of a strong, restrictive immune reaction to virus or tumor neoantigens. The diagnosis of PTCL, NOS is a frequent pitfall.
iT-LPD is a reactive proliferation often associated to reactive or neoplastic conditions. Criteria helpful for the diagnosis include a TdT+/CD3+ proliferation without atypia, no aberrant antigen expression, lack of clonality, no BM or mediastinal involvement, and no association with thymic epithelium
LV-HES is a reactive condition characterized by the presence of abnormal clonal, Th2 T-cell population with abnormal production of IL-5 and elevated IgE in serum. The differential diagnosis includes PTCL, NOS, TFH lymphomas and SS.
There are aggressive lymphomas, which might behave in an indolent fashion, at least for some time. Examples include T-ALL, TFH lymphoma and ENKTCL. The reason is not well-understood.
iTLPD-GI usually is confined to intestinal mucosae but may extend to all wall layers or be associated with a mesenteric LN involvement. These unusual features are still accepted as part of the disease.
Some cases of PD1+ T-LPD with low-grade features and intestinal tropism show PB, BM and/or skin dissemination at diagnosis or progress to aggressive TCL. In these cases, PD1 expression and higher proliferation (>10%) than iTLPD-GI may be used as differential markers. The panel proposed the term “indolent, systemic PD1+T-cell lymphoma/LPD” for better recognition and further analysis.
Most indolent B-cell lymphomas are well-defined entities in the WHO/ICC classifications such as DFL, MALT and leukemic non-nodal MCL.
Double-hit FL or a single atypical follicle with genetic alteration, are not well-characterized lesions. The prognostic impact of these alterations and/or their optimal therapeutic management warrant further analysis.
FCL of the lower female genital tract is a rare *BCL2*-R negative FCL, similar to PCFCL, expanding the group of extranodal *BCL2*-R negative FL entities. Despite the frequent presence of large cells, it is characterized by localized disease with low or no risk of dissemination or transformation.

The cases submitted also demonstrated which cases should not be considered indolent LPDs, despite a relative indolent clinical course, and this includes (1) EBV-positive LPDs, especially in immunosuppressed patients, (2) low-grade lymphomas requiring systemic treatment, and (3) lymphomas achieving complete remission after treatment.

## Supplementary Information

Below is the link to the electronic supplementary material.

Supplementary file 1 (DOCX 52.3 KB)

## Data Availability

Not applicable.
