# Supplementary material for: Exploring the boundaries between neoplastic and reactive lymphoproliferations: lymphoid neoplasms with indolent behavior and clonal lymphoproliferations—a report of the 2024 EA4HP/SH lymphoma workshop
Source: Virchows Arch. 2025 Jul 5;487(2):327–47. doi: 10.1007/s00428-025-04168-5 (PMC12391246; doi:10.1007/s00428-025-04168-5)
Supplement: Supplementary file 1 — (DOCX 52.3 KB) [file 428_2025_4168_MOESM1_ESM.docx]

**Supplemental Table 1. Clinicopathological features of 9 cases of primary cutaneous marginal zone lymphoproliferative disorder and**

**extranodal marginal zone lymphoma involving subcutaneous tissue or conjunctiva**

| **Case** | **Sex** | **Age** | **Clinical Presentation** | **Phenotype** | **Molecular** | **Panel diagnosis** | **Submitter** |
| --- | --- | --- | --- | --- | --- | --- | --- |
| LYWS-22* | M | 13 | Multiple cutaneous lesions, during 2 years | CD20+  CD10-, BCL6-,  IgG-Kappa | IGH clonal  TCR polyclonal | PC-MZLPD, class-switched rich in T-cells | A.R. Oskardottir  Rochester, USA |
| LYWS-47* | M | 61 | Patient with psoriatic arthritis treated with methotrexate, leflunomide etc. He developed nodular skin lesions in extremities treated with Rx. 1 year later recurred with increased large cells compatible with transformation. 2 years later progression with systemic disease | CD20+, BCL2+ CD10-, BCL6-,  IgG-kappa, EBV+  MiB1 <10%, at transformation 60-80%, CD30+, MUM1+, CD138+, MYC+ | FISH *MYC, BCL6, BCL2* wt  Mutations in: *DNMT3A*, *TET2* in BM (CHIP) and skin. At transformation *STAT3*  IGH/IGK, identical clonal population | EBV+PC-MZLPD, class-switched. Progression to large cells and transformation to plasmablastic lymphoma with dissemination | Lori Soma  City of hope, USA |
| LYWS-115 | F | 54 | Slowly growing preauricular erythematous plaque treated with Rx. The patient relapsed 4 times in the next 4 years. In the last skin relapsed a lymph node was involved. | CD20+, BCL2+, CD10-, BCL6-,  IgM+, kappa/lambda-  MiB1 < 10% | IGH identical clone in all biopsies | PC-MZLPD non-class-switched | A Mozos  Barcelona, Spain |
| LYWS-296 | M | 83 | Isolated subcutaneous nodule in scrotum  4.5x 4x 1.5 cm | CD20+, BCL2+  CD10-,BCL6-  MiB1 low | IGH clonal | PC-MZLPD | M. Abdulla  Uppsala, Sweden |
| LYWS-360 | F | 72 | Skin lesions. 4-5 scattered papules and patches on shoulder, chest, back and tight. No symptoms | CD20+, BCL2+  CD10-,BCL6-,  Kappa/lambda-  CXCR3+ | IGH clonal | PC-MZLPD, epidermotropic | J, Zhou  Arizona, USA |
| LYWS-463 | F | 49 | Swelling over neck skin | CD20+ BCL2+  CD123+ clusters of BPDC. Abundant T-cells  MiB1 15-20% | ND | PC-MZLPD | S. Epari  Mumbai, India |
| LYWS-241 | F | 65 | Skin lesion upper arm diagnosed as PC-MZLPD of class-switched type treated with Rx. Subsequently she developed a conjunctival lesion. | Skin: Kappa-IgG4+  Conjunctiva: CD20+ BCL2+ and also Kappa-IgG4+  Flow: 1.6% Kappa restricted B cells | IGH polyclonal.  No IGH gene comparison between the two lesions | Extranodal MZL, class-switched, IgG4+ | L. Qiu  Seattle, USA |
| LYWS-6 | F | 60 | Multiple PET-avid subcutaneous tissue masses in trunk. No other symptoms | CD10- BCL6+ in GC  IGA-lambda+  Equivocal BCL2+?  CD123+ clusters, EBER- | FISH*: BCL2, BCL6* wt  Mutations; *MYC (VAF:20%), ARID1A (13%), KMT2D (16%) and IRF4 (13%)* | Extranodal MZL  Class-switched | J. Staniforth  Cambridge, UK |
| LYWS-351 | F | 71 | A back subcutaneous mass of 6 months of duration | CD20+, BCL2+  MiB1 10-15%  CD10- BCL6- | IGH/IGK polyclonal  FISH: 18q/*MALT1* gains in 29% of nuclei Mutations: *BTG1* (VAFS: 27% and 25%), *BCL6* (7%), *MYD88*:wt | Extranodal MZL | L.K. Aftab,  Buffalo, USA |

LYWS: lymphoma workshop; FISH; fluorescence in situ hybridization; ND: not done; VAF: variant allelic frequency; F:female; M:male

Rx: patients treated with radiotherapy; PC-MZLPD: primary cutaneous marginal zone lymphoproliferative disorder *Case presented in the workshop

**Supplemental Table 2. Clinicopathological features of 4 cases with overlapping features between PC-MZLPD and PC-CD4+ T-cell LPD and 2 cases of primary cutaneous CD8+ T-cell lymphoproliferative disorder**

| **Case** | **Sex** | **Age** | **Clinical Presentation** | **Phenotype** | **Molecular** | **Panel diagnosis** | **Submitter** |
| --- | --- | --- | --- | --- | --- | --- | --- |
| LYWS-303* | M | 37 | Multiple painless lesions, left shoulder and upper back, 2 years  Tx excision lesions, CR, 2 years | CD20+, MUM1+  IgG4-Kappa  CD3+, CD4+, PD1+, ICOS+, CD7 partially loss. MIB1 low. | IGH clonal  TR clonal  NGS:no mutations | PC-MZLPD class-switched/PC-CD4+T-cell LPD | A. Vogelsberg  Tübingen, Germany |
| LYWS-192 | M | 80 | The patient had melanoma treated with vaccine and remission since 2000.  In 2023 a solitary itchy skin lesion rich in TFH cells. | CD20+, MUM1+  IgG and a subset IgG4  No light chain restriction  CD4+ infiltrate with a subset PD1+, ICOS+  Ki-67: 5-10% | IGH clonal  TR polyclonal | PC-MZLPD class-switched rich in TFH cells | I. Obiorah  Virginia, USA |
| LYWS-290 | M | 44 | Presented with 1.5 cm plaque in head. Asymptomatic after resection. | CD20+ 45%, CD3+ 55%  CD4+, PD1 20%  MUM1+, No light chain restriction | IGH clonal  TR clonal | PC-MZLPD / PC-CD4+ T-cell LPD | J. Szakonovi  Budapest, Hungary |
| LYWS-471 | M | 43 | One skin lesion in the forearm. 1-year follow-up with watch and wait CR.  PET- everything normal | CD20+ (B-cells), MUM1+,  IgG-Lambda+  T cells: CD3+, CD4+, PD1, focally ICOS+ | IGH clonal  TR clonal | PC-MZLPD class switched / PC-CD4+ T-cell LPD | R. Rolim  Lisboa, Portugal |
| LYWS-167 | M | 74 | CML history treated with Gleevec and Bosutinib. One scalp lesion mainly in subcutaneous tissue  5 years follow-up. CR | CD3+CD8+CD5+CD2+CD7weak | TR clonal | PC-CD8+ T-cell LPD | R. Shao  Florida, USA |
| LYWS-229 | M | 41 | Helix ear lesion,  Typical example | CD3+CD8+  CD2/CD5 diminished  CD68+ golgi like  MiB1<10% | TR clonal | PC-CD8+ T-cell LPD | T. Irwin,  Portland, Oregon |

LYWS: lymphoma workshop; F:female; M:male; PC-MZLPD: primary cutaneous marginal zone lymphoproliferative disorder *Case presented in the workshop

PC-CD4+ T-cell LPD: primary cutaneous CD4+ small/medium T cell lymphoproliferative disorder; TFH: T-follicular helper cells; Tx: treatment;

CR: complete remission; TR: T cell receptor gene rearrangement; IGH: Immunoglobulin heavy chain gene. NGS: next generation sequencing.

**Supplemental Table 3. Clinicopathological features of 11 cases of Indolent T/B-lymphoblastic proliferation and mimickers**

| **Case** | **Sex** | **Age** | **Clinical Presentation** | **Phenotype** | **Molecular** | **Panel diagnosis** | **Submitter** |
| --- | --- | --- | --- | --- | --- | --- | --- |
| LYWS-246* | F | 86 | 4 cm parotid tumor. Follow-up 16 months CR. | CD3+CD4+CD8+TDt+CD10+ CD1A+ MIB1>95% | TR polyclonal | iT-LBP in parotid gland with acinar cell carcinoma | F. Pesce  Marseille, France |
| LYWS-60 | M | 46 | 2016 Right iliac lymphadenopathy. FL treated with R-bendamustin-  2017 PET showed progressed disease. New LN biopsy | FL:CD20+, CD23+, BCL2+, BCL6+, CD10-  Multifocal scattered TDT+ surrounding the follicles | FACS:42% clonal B-population  8.8% T-cells TDT+CD4+CD8+  FISH:*BCL2/BCL6* wt | iT-LBP with Follicular lymphoma | Y. Ju  University Southern California, USA |
| LYWS-74 | M | 40 | Left parotid tumor 4x3x3.5 cm  The lymphoid cells disseminated.  follow-up unremarkable | Tdt+CD3+CD5+BF1-CD10+CD1a+CD99+  MIB1 high | ND | iT-LBP in parotid gland with acinar cell carcinoma | C.L. Cheng  Singapore |
| LYWS-117 | M | 35 | Clinical history of Langerhans histiocytosis. PET-CT revealed high FDG uptake in tonsils. | TDT+CD3+ | ND | iT-LBP in tonsillar palatina | A.Y. Altay  Istanbul, Turkey |
| LYWS-183 | F | 53 | lymphadenopathy | FACS 11% of immature T-cells: CD1+, TDT+, CD10+/-, CD34- | TR polyclonal | iT-LBP in ovarian metastasis | D.S. Solima  Qatar |
| LYWS-266 | M | 40 | 6x4 cm mass in left psoas discovered by accident | CD3+,CD4+,CD8+,CD1a+,CD10+,TDT+  MiB1 90% | ND | iT-LBP with Castleman disease | P. Dartigues  Gustav Roussy, Paris |
| LYWS-315 | F | 35 | Autoimmune disorder. 7.5 cm isolated neck mass developed in 6 months. | CD3+,CD1a+,CD4+,CD8+,TDT+, CD10-  Mib1 90% | TR polyclonal  IGH polyclonal | iT-LBP with FDC sarcoma and Castleman disease | A.E. Quesada  MD Anderson, Houston, USA |
| LYWS-373 | F | 48 | Bilateral axillary LN | CD20+PAX5+ TDT+CD10+BCL6- | TR polylclonal  IGH polyclonal | iB-LBP | P. Devi  Uni Penn, USA |
| LYWS-35 | M | 48 | Mediastinal mass 8x7.8x8.1 | CD34- TDT+ CD1a CD4/CD8+ | TR polyclonal | Residual thymus with NEC mimicking iT-LBP | S. Bunting  Cleveland clinic  Florida, USA |
| LYWS-215 | F | 39 | Mediastinal mass 3 cm | CD3+ TDT+ CD1a+ CD4/CD8+  CD10/LMO2/CD34 neg | TR polyclonal | True thymic hyperplasia | G.A. Croci  Milano, Italy |
| LYWS-196 | F | 73 | Large cervical LN followed by extensive lymphadenopathy one year later | CD3+,dim CD5, CD4, CD2, TDT, CD99 and CD1a. CD7-, CD8-, CD10-  MiB1: 80-90% | TR monoclonal  Flow: CD3+, CD2+, CD5+, CD7+, CD99+, CD38+ CD13 and CD33 (subset) | T-LBL with protracted presentation | Y. Jin  Florida, USA |

LYWS: lymphoma workshop; F:female; M:male; PC-MZLPD: primary cutaneous marginal zone lymphoproliferative disorder *Case presented in the workshop

PC-CD4+ T-cell LPD: primary cutaneous CD4+ small/medium T cell lymphoproliferative disorder; TFH: T-follicular helper cells; Tx: treatment;

CR: complete remission; TR: T cell receptor gene rearrangement; IGH: Immunoglobulin heavy chain gene. NGS: next generation sequencing.

**Supplemental Table 4. Clinicopathological features of 8 cases of “other indolent” T-cell proliferations**

| **Case** | **Sex** | **Age** | **Clinical Presentation** | **Phenotype** | **Molecular** | **Panel diagnosis** | **Submitter** |
| --- | --- | --- | --- | --- | --- | --- | --- |
| LYWS-344* | F | 62 | In 2020 poorly differentiated squamous cell carcinoma of oropharynx, HPV and EBV negative. Multiple hypermetabolic deposits in PET/CT in muscle suggestive of metastasis | CD3+ CD8+ CD2+ CCD7+ TIA1+ GRzB+  CD5- and BF1-/+  Ki-67 75%  Flow CD8+ memory cells (CD27+, CD28+, CD62L-, CCR7-, CD45RA-, CD45RO+, CD57-) | TR monoclonal, identical in all samples  NGS: no mutations | Reactive clonal CD8+ T-cell LPD in muscles | M. Gomez-Tena  Bellvitge, Barcelona |
| LYWS-276 | M | 21 | Celiac disease, presented with meningitis  Spinal fluid HHV7 viral infection.  Responded to steroids, 3 years later asymptomatic | CD4-/CD8- T cells lacking CD7 and TRVß 7.1 restriction | TR monoclonal  ALPS tests were negative | CD4/CD8 negative T-cell LPD occurring in a dysimmune setting | M. Pizzi  Padua, Italy |
| LYWS-330 | M | 34 | The patient presented with acute abdominal pain due to spontaneous splenic rupture. | CD3+ CD8+ CD5+ TIA1+ GRB+ BF1+  EBER/CD20+ cells | TR monoclonal in liver and spleen  IGH polyclonal | Clonal CD8+ T-cell LPD secondary to IM | L. Quintanilla-Fend  Tübingen, Germany |
| LYWS-380 | M | 31 | 2005 Leg edema with eosinophilia. 11 300 eos. FACS: T-cells normal  The patient was diagnosed with HES treated with Nucala  2015: HES  2017: BM smear 35% eosinophils, FACS 23% T cells of which 50% aberrant phenotype CD5+++, CD3-, CD2+, CD4+. A diagnosis of LV-HES was rendered.  2017-2020 PB showed leukemic picture with atypical cells. Skin involvement  2020: Diagnosis of PTCL , NOS was made  The patient was treated with Chx followed by ASCT. | Flow: CD5+++, CD3-, CD2+, CD4+, CD8-, CD38-, CD57-, CD16- | TR monoclonal in skin, PB and BM.  Normal karyotype  *FIP1L1, PDGFRa/b FGRG1* and *BCR-ABL* negative.  *STAT3* mutation | Lymphocyte variant-Hypereosinophilic syndrome (LV-HES) | A.Kwiecinska  Karolinska, Sweden |
| LYWS-212 | F | 79 | Lung adenocarcinoma and unexplained eosinophilia since 1960 | Flow: CD3-, CD7-, CD5+++, CD4+ | TR monoclonal in blood and BM.  Normal karyotype  *FIP1L1, PDGFRa/b FGRG1* and *BCR-ABL*, *JAK2* negative.  No *KIT* mutations  NGS: no mutations | Lymphocyte variant-Hypereosinophilic syndrome (LV-HES) | D.I. Laczko  Uni Penn, Philadelphia, USA |
| LYWS-361 | F | 63 | Mass in the right groin for 3 months. Imaging revealed several enlarged LN.  The patient has not received treatment and is asymptomatic 73 mo of follow-up | CD20+ hyperplastic follicles surrounded by clusters of CD3+CD4+ T-cells, PD1+, ICOS+, CD10+, BCL6+. | TR monoclonal  IGH polyclonal  NGS: mutations in TET2, RHOA, BARD1 and ATR | PTGC like TFH lymphoma with indolent behaviour | Y. Zheng,  Beijing, China |
| LYWS-408 | M | 52 | Born in Guinea Equatorial, with history of Thalassemia minor, mild neutropenia and epilepsy.  2022 presented with pruritic lesions.  2020. 2022, 2023 skin biopsies | CD20+ nodules surrounded by CD3+ CD4+ CD5+, PD1+, CXCL13+, BCL6+, CD10+, GATA3+, BF1+, CD7- and low proliferation | TR monoclonal identical in all samples  NGS: not performed | PD1+ clonal skin proliferation, favors Sezary syndrome | L. Colomo, Barcelona, Spain |
| LYWS-449 | F | 76 | 2019 initiate with skin lesions  2022 Patient with extensive erythroderma treated with UVB (no response)  2023 worsening of skin lesions a biopsy diagnosed as PTCL, no mycosis fungoides. Stage IV with PB and BM  Undergoing systemic chemotherpy. | CD3+, CD4+, CD5+, PD1+, CD7-, CD2dim. CD30+ (small subset), P53-  Flow: abnormal T-cell population (74%). CD3+, CD4+, CD8-, CD5+, CD7dim, CD26+, TCRb1+ | TR monoclonal identical in multiple samples.  NGS: *ARID5B* (VAF:13%); *CHD1* (VAF 16%); *FAT1* (VAF 16%) | PD1+ clonal leukemic, skin, LN, favors Sezary syndrome | G. Rassidakis  Stockholm, Sweden |

LYWS: lymphoma workshop; F:female; M:male; *Case presented in the workshop; TFH: T-follicular helper cells; Tx: treatment; PB: peripheral blood

Chx: chemotherapy; TR: T cell receptor gene rearrangement; IGH: Immunoglobulin heavy chain gene. NGS: next generation sequencing.

PTCL: peripheral T-cell lymphoma; BM: bone marrow; LN: lymph node; PTC: progressive transformation of germinal centers; ALPS: autoimmune lymphoid

proliferative syndrome; ASCT: autologous stem cell transplantation; LPD: lymphoproliferative disorder

**Supplemental Table 5. Clinicopathological features of 6 cases of EBV+ B, T or NK cell lymphoproliferations**

| **Case** | **Sex** | **Age** | **Clinical Presentation** | **Phenotype** | **Molecular** | **Panel diagnosis** | **Submitter** |
| --- | --- | --- | --- | --- | --- | --- | --- |
| LYWS-340 | F | 44 | 2020. Sore throat and enlarged cervical lymph nodes. Retrospectively the patient had a palate biopsy in 2017 with the same EBV+CD56+ infiltrate.  2021 a LN biopsy was infiltrated she received DDGP regimen followed by ASCT  2024 CR | CD3+, CD2+, CD7+, CD56+ TIA1+, GrB+, perforin+  EBER+, MIB1 60% | ND | Extranodal NK/T cell lymphoma, nasal type | T. Pikivaca  Zagreb, Croatia |
| LYWS-293 | F | 32 | Since 2017 recurrent fever and nasal obstruction with rhinorrhea. History 4 years before of an acute EBV infection  2019 FDG/PET documented intense uptake in nasal cavities and nasopharynx | CD3+CD8+ CD56-  EBER+ | TR monoclonal in 2023  Whole genome revealed a *RIPK1* germline mutation | Extranodal NK/T cell lymphoma, nasal type with indolent behaviour | L. Bonngiovanni  Milan, Italy |
| LYWS-250 | M | 23 | Hispanic patient with right lip lesion. He developed persistent fever and facial swelling, blisters in abdomen and tight which rapidly progressed to black necrotic ulcers. No mosquito allergy. High EBV viral load.  Gastric perforation also infiltrated. | CD3+ CD8+ CD5+ TIA1+ GRB+ BF1+  EBER/CD20+ cells | TR monoclonal in skin and BM  DDX3X 52%, GNAS 46% and PTPN1 50%  Uncertain significance | CAEBV disease, progressed to a CD8+ PTCL. | P. Devi  Uni Penn, USA |
| LYWS-61 | M | 30 | HIV+ diagnosed 4 months prior to presentation, count of 43 CD4/mm3. Presented with progressive mental and cognitive decline, B symptoms. MRI necrotic lesion in right temporal and left occipital lobes | CD20+ cells surrounded by CD3+ cells. Few EBER+ cells | IGH/IGK monoclonal | EBV+ Polymorphic monoclonal B cell LPD in HIV+, recovered after ART therapy | D. Even Fridrich  Tamat Gan, Israel |
| LYWS-363 | M | 53 | Long history of Tabacco dependence and COPD with left-sided neck and back pain. CT scan large pleural effusion | CD30+, PAX5+, rare CD20+, CD79a, MUM1 and CD138 weak and some. EBER+ and LMP1+ | FISH: *BCL2, BCL6* and *MYC* pending  NGS pending | Diffuse large B-cell lymphoma with chronic inflammation | A.Toklu  Indiana, USA |
| LYWS-138 | M | 35 | HIV+ under antiretroviral therapy with B symptoms and lymphadenopathy and mediastinal mass. Resistant to multiple therapies, Died of COVID | EBER+, RS: partial CD30+ CD15- CD20+ PAX5+ MUM1+ | IGH polyclonal  TR polyclonal | EBV+ polymorphic LPD in HIV setting | T. Lakic  Serbia |

**Supplemental Table 6. Clinicopathological features of 14 cases submitted with the diagnosis of iT/NK-LPD of the GI.**

| **Indolent T/NK-LPD of the GI with typical features** | | | | | | | | | |
| --- | --- | --- | --- | --- | --- | --- | --- | --- | --- |
| **Case** | **Age** | **Sex** | **Clinical Presentation** | **Site of involvement** | **Phenotype** | **Clonality** | **Molecular** | **Panel diagnosis** | **Submitter** |
| LYWS-24 | 67 | F | Pain and weight loss | Duodeno-jejunal | CD4- CD8-  Ki67 <5%  **PD1 (w)+** | TR monoclonal | *FAT1* variant | **iTLPD-GI** | J. Karrs, Lebanon |
| LYWS-211 | 42 | M | Past of malabsorption | Small bowel | CD8+ CD4- CD20-  Ki67 <5%  **PD1-** | TR monoclonal | ND | **iTLPD-GI** **treated with MTX and CTX for 10y with secondary development of DLBCL EBV-** | N. Vidal-Robau, Barcelona, Spain |
| LYWS-328 | 16 | M | diarrhoea | Small bowel | CD4- CD8- CD20+  Ki67 <5% | TR monoclonal | No mutation | **iTLPD-GI** | D. Nann, Tübingen, Germany |
| LYWS-349 | 30 | M | Diarrhoea and auto-immune enteropathy context | Duodeno-jejunal | CD8+ CD4-  Ki67 <5% | TR monoclonal | *IRF8* splice region variant  *STAT5b* missense VUS | **iTLPD-GI** | E. Hookway, London, UK |
| LYWS-454 | 60 | F | Crohn disease context | Small bowel | CD8+ CD4- CD20+  Ki67 <5%  **PD1-** | TR monoclonal | ND | **iTLPD-GI** | F. A. Green, London, UK |
| LYWS-79 | F | 35 | Abdominal pain, diarrhoea and B symptoms  Crohn disease | Small bowel | NK phenotype  CD20- | TR polyclonal | *JAK3* variant | **iNK-LPD of GI** | M. Shi, Rochester, USA |
| **Indolent T/NK LPD of the GI with unusual presentation** | | | | | | | | | |
| LYWS-96 | F | 37 | Abdominal pain diarrhoea weight loss | Duodeno-jejunal  Mesenteric LN and SMG | CD4+ CD20-  KI67<5%  PD1- | ND | ND | **iTLPD-GI with wall layers extension** | C. Abou-Seif, Clayton, USA |
| LYWS-253 | M | 62 | Malabsorption symptoms | Duodeno-jejunal | CD4+ CD20-  KI67<5%  PD1- | TR monoclonal | ND | **iTLPD-GI with wall layers extension** | L. Goh, Singapore |
| LYWS-256 | M | 66 | Chronic diarrhoea | Small bowel  Mesenteric LN | CD8+ CD20-  KI67<5%  PD1- | TR monoclonal | ND | **iTLPD-GI with wall layers extension** | F. Climent, Barcelona, Spain |
| LYWS-461* | M | 74 | Melena revealing a single polypoid lesion | jejunum | CD8+ CD20-  KI67<5%  PD1- | TR monoclonal | *DNMT3A* variant | **iTLPD-GI with wall layers extension** | P. Gaulard, Paris, France |
| **Other intestinal T-LPD/lymphoma** | | | | | | | | | |
| LYWS-337 | M | 40 | Incidental mesenteric LN presentation | LN, BM/PB and intestinal involvement. | CD4+  Ki67 10%  **PD1+** | TR monoclonal | *STAT3* variant | **Indolent systemic CD4+ T cell lymphoma/LPD** | L. Mescam, Marseille, France |
| LYWS-364* | M | 39 | Routine gastrodudenal endoscopy retroperitoneal LN and SMG | Gastroduodenal, LN, SMG, PB and BM involvement | CD4+, aberrant CD20+  Ki67>20%  **PD1+** | TR monoclonal | *JAK1* and *TET2* VUS variant | **Indolent systemic CD4+ T cell lymphoma/LPD progressing to a systemic aggressive T-cell lymphoma, NOS** | E. M. Gerhard-Hartmann, Würzburg, Germany |
| LYWS-376 | M | 67 | Swollen epiglottis with past history of vasculitis and lymphadenopathy since 10 y | Epiglottis with LN and skin involvement. | CD4+,  Ki67 10%  **PD1+** | TR monoclonal | No alterations | **Indolent systemic CD4+ T cell lymphoma/LPD** | P. Cervera, Paris, France |
| **Reactive T cell infiltrates** | | | | | | | | | |
| LYWS-191 | 37 | F | History of Crohn disease and autoimmune hepatitis since 12-year-old | Abdominal pain and recurrent episode of nausea, vomiting | B and T- cell aggregates in the submucosae  ki67 <10% | Oligoclonal T cell expansion | ND | **Oligoclonal T cell expansion in an inflammatory bowel disease** | M. Moore,  Charlottesville, USA |

BM: bone marrow; CTX: cyclosporine; LPD: lymphoproliferation disorder; LYWS: lymphoma workshop; LN: Lymph node; MTX: methotrexate; ND not done; PB: peripheral blood; TR: TCR rearranged; SMG: splenomegaly; TR: T-cell receptor genes; VUS: variant of uncertain significance; *Case presented in the workshop

**Supplemental Table 7. Clinicopathological features of 6 cases submitted with the diagnosis of duodenal follicular lymphoma.**

| **Case** | **Age** | **Sex** | **Clinical Presentation** | **Site of involvement** | **Phenotype** | **FISH** | **Molecular** | **Panel diagnosis** | **Submitter** |
| --- | --- | --- | --- | --- | --- | --- | --- | --- | --- |
| LYWS-165 | 36 | M | Abdominal pain due to ileal ulcer | Duodenal and ileum involvement | CD20+ CD10+ BCL2+  FDC CD21 + | ND | ND | **DFL** | R. Shao, Orlando, USA |
| LYWS-322 | 65 | M | Constipation due to small bowel obstruction | Duodenal | CD20+ CD10+ BCL2+  FDC CD21 + | *BCL2*-R positive | *CREBBP HVCN1 KMT2D* | **DFL** | R. Gonta, Houston, USA |
| LYWS-378 | 51 | M | Incidental  (esophageal varices rupture in cirrhosis context) | Duodenal | CD20+ CD10+ BCL2+  FDC CD21 + | ND | ND | **DFL** | L. Goh, Singapore |
| LYWS-404 | 60 | F | Gastro-oesophageal reflux | Duodenal | CD20+ CD10+ BCL2+  FDC CD21 + | ND | ND | **DFL** | B. Burroni, Paris, France |
| LYWS-462 | 55 | F | Abdominal pain | Duodenal | CD20+ CD10+ BCL2+  FDC CD21 + | ND | ND | **DFL** | M. Medani, London, UK |
| LYWS-396 | 44 | M | Symptomatic dyspepsia | Duodenal | CD20+ CD10- BCL6- BCL2+ CD138 weak  No FDC network | No *BCL2*-R | *CD79b* variant | **MALT lymphoma** | U. Sakhadeo, Mumbai, India |

*BCL2*-R: BCL2 rearranged; DFL duodenal follicular lymphoma; FDC follicular dendritic cell; LYWS: lymphoma workshop; ND not done;

**Supplemental Table 8. Clinicopathological features of 11 cases of B-cell LPD/lymphomas with low grade or indolent features**

| **Indolent non-duodenal FL/FL-related lesions** | | | | | | | | | |
| --- | --- | --- | --- | --- | --- | --- | --- | --- | --- |
| **Case** | **Age** | **Sex** | **Clinical Presentation** | **Site of involvement** | **Phenotype** | **FISH** | **Molecular** | **Panel diagnosis** | **Submitter** |
| LYWS-100 | 45 | F | Neck node and skin thickening with erythema | Skin and LN | CD20+ CD10- BCL2+ BCL6+ LMO2+ | *BCL2*-R positive | ND | **Skin involvement by a conventional FL grade 1-2** | T. Shet, Mumbai, India |
| LYWS-317* | 22 | F | Uterine mass without LN | Cervico-uterine | CD20+ CD10- BCL2- BCL6+  Ki67 50-60% | No *BCL2*-R  No *BCL6*-R | *TNFRSF14*, *S1PR2*, *IGLL5*  *and EZH2* variants | **Follicle center lymphoma of the lower female genital tract.** | V. Leventaki, Houston, USA |
| LYWS-338* | 45 | F | Weight loss without LN | Mass tongue and subsequent submandibular involvement 9 months later | CD20+ CD10+ BCL6+ BCL2+ CD23+ Ki67 variable | *BCL2*-R positive *MYC*-R positive | ND | **Extranodal FL with *BCL2-*R and *MYC*-R** | M. Recuero-Pradillo, Toledo, Spain |
| **Isolated atypical follicle with genomic alteration** | | | | | | | | | |
| LYWS-38* | 46 | F | Sentinel axillary LN in a context of breast DCIS | Isolated atypical follicle in a LN | CD20+ CD10+ MYC+ LMO2+ LEF1+ BCL6+ BCL2-  Ki67 95% | *MYC*-R positive  No *BCL2*-R  *No BCL6*-R | ND | Incipient Burkitt lymphoma | M. Yabe,  New York, USA |
| LYWS-319 | 17 | M | Isolated thigh lymphadenopathy | Isolated atypical follicle in a LN | CD20+ CD10+ BCL6+ BCL2-  Ki67 40% with lack of polarization | *No BCL2*-R  *No BCL6*-R  No *IRF4*-R | ND | Atypical follicle with *BCL6* polysomy | J. Sidhu,  New York, USA |
| **Single examples of well-defined low-grade B-cell lymphomas/LPD** | | | | | | | | | |
| LYWS-358 | 60 | M | Abdominal pain | Stomach  (no leukemic presentation) | CD20+ CD5+- Cyclin D1+ SOX11-  Ki67 10% + | *CCND1::*IGH positive *BIRC::MALT1 negative* | ND | **Indolent MCL**  **(SOX11 negative non nodal MCL)** | E.F. Mason, Nashville, USA |
| LYWS-386 | 71 | M | SMG thrombopenia monocytopenia and circulating hairy cells  Context of Rosai Dorfman disease involving skin | Biopsy and aspirate BM | Biopsy : CD20+ annexin+ TBX21+ cyclin D1- BRAF V600E- TRAP+  Flow : CD11c+ CD19+ CD20+ CD22+ CD25+  CD103+, CD200+, HLA-DR+ and kappa restriction CD5- CD10- CD43- CD23- CD123- | Normal Karyotype | *MAP2K1* and *CREBBP* variants (no *BRAF* alterations) | **CD123- *BRAF*- Hairy cell leukemia** | B. Thakral, Houston, USA |
| LYWS-407 | 73 | NA | Intraocular lesion | Eye with PB involvement | CD20+ CD5+ cyclin D1- SOX11-  kappa restriction  Ki67 30% | *CCND1::*IGH positive | B cell clone  *MYD88 S219C* variant | **Leukemic non nodal MCL with plasmocytic differentiation** | I. Blázquez Muñoz, Barcelona, Spain |
| LYWS-419 | 82 | F | Nausea and diarrhea due to infectious gastroenteritis complicated by pneumonia and hemolytic anemia | PB | CD20+ CD22+ CD5dim+ CD10- CD19+ CD23- CD103- CD25- CD38+ kappa restriction | ND | ND | **Cold agglutinin disease/ Primary cold agglutinin disease** | L. Carrillo, Durham, USA |
| LYWS-420 | 68 | M | CT scan finding of lung nodule | Lung | CD20+ CD10- BCL6- BCL2+ with  lambda >>kappa  ki67 5% | *MALT1*-R negative | B cell clone  *No MYD88* L265P | **MALT lymphoma** | P. Cervera, Paris, France |
| LYWS-460 | 41 | M | Abdominal pain colon wall thick polyadenopathies | LN | CD20+ PAX5w CD10- MUM1+ TBX21+  kappa >> lambda  ki67 30%  CD21 distorted  reactive TFH cells expansion | ND | B cell clone  *TNFRS14* variant | **TFH rich NMZL** | S. Epari, Mumbai, India |

*BCL2*- or *BCL6*-R: *BCL2*- or *BCL6*-R rearranged; BM: bone marrow; DCIS : ductal carcinoma in situ; FL : follicular lymphoma; *IRF4*-R: *IRF4* rearranged. LPD: lymphoproliferation disorder; LYWS: lymphoma workshop; LN: Lymph node; *MALT1*-R: *MALT1* rearranged; MALT lymphoma: mucosa-assisted lymphoid tissue lymphoma; MCL: Mantle cell lymphoma; *MYC*-R: *MYC* rearranged; NMZL: nodal marginal zone lymphoma; ND not done; PB: peripheral blood; TFH : T follicular helper; SMG: splenomegaly.
